# Views of knowledge users on recurrent miscarriage services and supports in the Republic of Ireland: a qualitative interview study

**DOI:** 10.1136/bmjopen-2024-094753

**Published:** 2025-04-10

**Authors:** Marita Hennessy, Rebecca Dennehy, Karen Matvienko-Sikar, Ria O’Sullivan-Lago, Jennifer Uí Dhúbhgáin, Con Lucey, Keelin O’Donoghue

**Affiliations:** 1 Pregnancy Loss Research Group, Department of Obstetrics & Gynaecology, University College Cork, Cork, Ireland; 2 INFANT Research Centre, University College Cork, Cork, Ireland; 3 School of Public Health, University College Cork, Cork, Ireland; 4 Waterstone Clinic, Cork, Ireland; 5 RE:CURRENT Research Advisory Group, Pregnancy Loss Research Group, Department of Obstetrics and Gynaecology, University College Cork, Cork, Ireland; 6 Miscarriage Association of Ireland, Dublin, Ireland

**Keywords:** QUALITATIVE RESEARCH, Reproductive medicine, Health Services, Person-Centered Care, Pregnancy

## Abstract

**Abstract:**

**Objectives:**

Women and men/partners who experience miscarriage often report poor care experiences within health services around the time of miscarriage and beyond; less is known about recurrent miscarriage (RM) care. Research is needed to explore the potential targets for improvement, in addition to identifying factors that support or hinder service improvement efforts and the implementation and/or sustainment of desired models of RM care. This study aimed to explore the views of knowledge users regarding RM services and supports; specifically: (a) practices and experiences and (b) facilitators and barriers to providing desired services and supports.

**Study design:**

We adopted a qualitative study design underpinned by constructivism, incorporating semistructured interviews. Data were analysed using reflexive thematic analysis.

**Setting:**

Participants were recruited across the Republic of Ireland, incorporating perspectives from different geographical areas, hospital types and RM services.

**Participants:**

We interviewed 13 women and 7 men/partners who had experienced ≥2 consecutive miscarriages, and 42 people involved in the delivery and/or management of RM services and supports, between June 2020 and February 2021.

**Results:**

We generated three themes from the data: (1) dedicated staff; (2) dedicated space and time and (3) dedicated funding and support—prioritise RM. Our analysis supports the need for a standardised, dedicated and adequately resourced and supported service. One in which people experiencing RM are offered appropriate, individualised, timely and accessible care and support—beginning following the first miscarriage, and following a graded model. Implementation requires several multilevel actions, including prioritising RM care, adequately funding and resourcing services, enhancing health professional education and support, care coordination within and between hospitals and primary care and improving public awareness of, and addressing stigma surrounding, miscarriage.

**Conclusions:**

Our analysis provides context to ‘good’ and ‘poor’ care experiences and identifies what facilitators and barriers exist to affecting change in RM care within healthcare and broader systems. In light of recent debates regarding how best to deliver RM care, and changing international guidelines, this work provides timely and important knowledge that should be harnessed to inform service improvement efforts in the Republic of Ireland and beyond.

**STRENGTHS AND LIMITATIONS OF THIS STUDY**

- The inclusion of a diverse range of perspectives—including people with lived and professional expertise—is a key strength of this study.
- We situated our evaluation of services within broader healthcare, social and political contexts.
- A limitation of our study is the lack of ethnic/cultural diversity within the sample.

## Introduction

 Miscarriage is generally defined as the spontaneous loss of a pregnancy before it reaches viability.^1^ The population prevalence of women who have had one, two, or three or more miscarriages is 10.8%, 1.9% and 0.7%, respectively.^1^ Miscarriage has physical, psychological and economic impacts,^1^ and research suggests that symptoms of perinatal grief and depression can persist long after a miscarriage, particularly for women who are dissatisfied with healthcare services.^2^ Negative psychological impacts, including grief, depression, anxiety, stress and post-traumatic stress disorder, can be amplified for people with recurrent miscarriage (RM).^3^,^7^

While much research has explored women’s, and increasingly men’s, lived experiences of miscarriage and miscarriage care in the first trimester,^8^,^18^ there has been less focus specifically on recurrent first trimester miscarriage, though research is increasing.^419^,^25^ Research has tended to focus on perinatal death, and this is also the case regarding health professionals’ experiences.^26^,^28^ Few studies have explored health professionals’ experiences of, and the facilitators and barriers to, providing care following first trimester miscarriage.^29^,^31^ Recurring themes across the aforementioned literature include deficits regarding communication and information, hospital environments (eg, shared spaces for pregnancy loss and maternity care) and supportive and follow-up care, as well as the lack of training, supports and resources for health professionals. Such issues can exacerbate distress for people who experience pregnancy loss.^10^ There is much debate about how to organise and provide care for people who experience RM, with consensus growing for a graded model in which women are offered appropriate, individualised support following their first and subsequent miscarriages.^32^ This includes offering people healthcare advice and support after one miscarriage, care in a nurse or midwife-led clinic after two miscarriages and care in a medical consultant-led clinic after three miscarriages, in line with the Lancet Commission’s recommendations.^32^ Research is needed to explore the potential targets for improvement of RM care,^17^ in addition to identifying factors that support or hinder service improvement efforts and the implementation and/or sustainment of desired models of care.

The aim of this research is to explore views of people with professional and lived experience regarding RM services and support. Specifically, we aim to examine existing (a) practices and experiences and (b) facilitators and barriers to providing the desired services and supports. Building on our initial exploration of how RM is defined,^33^ which has implications for when and how people can access support for RM (including investigations, treatments and care in subsequent pregnancy), this paper examines what such services and supports should encompass.

## Methods

This study is part of a larger project titled RE:CURRENT (REcurrent miscarriage: evaluating CURRENT services),^34^ and is reported in line with Standards for Reporting Qualitative Research.^35^

We employed a qualitative study design, underpinned by constructivism and incorporating semistructured interviews and reflexive thematic analysis, to facilitate an in-depth exploration of the views and experiences of women and men with lived experience of RM and those involved in the provision of services and supports. Aligned with this, we acknowledge that knowledge is socially produced, and the role that researchers play in co-creating knowledge and meaning.^36^ We adopted a critical qualitative inquiry approach within our study, underpinned by social justice,^37^ as we aimed to interpret people’s views and experiences of RM care while also trying to affect change in service provision. Throughout the study, we attended to wider organisational contexts and systems during data generation and analysis, given that power operates at multiple levels to influence action around improving healthcare experiences, including power held (or indeed withheld) within and across service users, staff, teams and broader hospital and healthcare systems and structures.^38^

The multidisciplinary research team included a woman and man with lived experience of RM (JUD, CL), three social scientists (RD, SM, ROSL), a public health/health services researcher (MH), a health psychologist (KM-S) and an obstetrician and maternal fetalmedicine subspecialist (KOD); all with an interest in enhancing care experiences and outcomes for people who experience pregnancy loss, including partners, and with lived experience, research or clinical expertise in RM and/or maternal and child health.

### Ethical considerations

Informed consent was obtained from participants electronically in advance, and verbal consent was clarified before interview commencement and throughout.^39^ Members of the research team were experienced in qualitative data collection on sensitive topics, including pregnancy loss. Distress protocols were in place for participants, researchers and the professional transcriptionist; these are outlined elsewhere.^40^

### Patient and public involvement

Members of the RE:CURRENT Research Advisory Group (two of whom are co-authors) reviewed, piloted and refined recruitment materials and topic guides, recruited participants through their networks and discussed coding and provisional themes with the research team during the analysis.

### Setting and participants

A purposive sampling strategy, supplemented with snowballing techniques, was used to recruit people with knowledge and experience of delivering and/or using RM services and supports in the Republic of Ireland where—at the time of the research—RM was typically defined as three or more consecutive miscarriages.^33^ Women and men/partners aged over 18 years and who had two or more consecutive first-trimester miscarriages (to ensure that we gained insights into the experiences of those who met the referral criteria for RM services and those who did not, also reflecting the varying definitions of RM internationally^41^), with the most recent loss occurring 6–24 months prior to study participation were eligible to participate. The latter was to allow sufficient time since the most recent miscarriage while capturing relatively recent experiences of services and supports. We also aimed to ensure that perspectives from various hospital groups (N=6), and tertiary, regional and peripheral maternity units (N=19) across the Republic of Ireland were included. Additional information about our study context is available in online supplemental file 1.

Participants were identified and recruited through the professional, collegial and support networks of the research team and RE:CURRENT Research Advisory Group; this included recruitment of people known to the research team or Advisory Group members in personal or professional contexts including hospitals, support organisations and professional bodies. Social media was used to enhance the recruitment of women and men/partners with RM in later phases of the study. We ceased data collection when there was sufficient depth and variation in the data.^42^

### Data generation

RD and M conducted semi-structured virtual interviews with participants via Zoom,^43^ with the option of continuing interviews via telephone in the event of dropped/poor connection, between June 2020 and February 2021. Videoconferencing has been used successfully in qualitative data collection, providing advantages over telephone interviewing in that it maintains the visibility of the non-verbal cues and interactions that serve to build rapport between interviewer and participant and promote meaningful discussion.^44^ All interviews were audio recorded.

Two topic guides were used to structure discussions, one specific to people with lived experience, and one for those involved in RM service and/or support provision (see onlinesupplemental files 2  3, respectively). These were informed by the extant literature and discussions with the RE:CURRENT Research Advisory Group. Topics covered management of RM, knowledge and understanding, impact and support, and recommendations. In addition, providers were engaged in discussion about how RM is defined and the structure of RM care, while people with lived experience were asked about expectations of pregnancy before loss and subsequent pregnancies. Topic guides were piloted, and then refined, and reviewed throughout the data collection process. Field notes were written following each interview to inform the ongoing development of the study, researcher reflexivity, and analysis.

### Data analysis

Audio data were transcribed verbatim by a professional transcriptionist. Transcripts were checked for accuracy, anonymised and imported into NVivo V.12.^45^ Using reflexive thematic analysis, a rigorous iterative process of data familiarisation, data coding, theme development and revision, refinement and write-up was undertaken^36^; see further details in online supplemental file 4. We analysed the data inductively, and data from women and men with lived experience of RM and those involved in the provision of services and supports together. During data generation and analysis, we observed similarities within the data from both groupings; therefore, we analysed them together to provide a multifaceted understanding of RM services and supports. All members of the research team were involved in analysis with RD, MH, SM, KM-S and ROSL meeting frequently to share and discuss their reflections, interpretations and positionalities to enhance theme development. Preliminary themes were presented to members of the RE:CURRENT Research Advisory Group (including JUD and CL) for feedback before being finalised.

## Results

Between June 2020 and February 2021, we interviewed 42 people involved in the management/delivery of services and supports, and 20 people (13 women, 7 men) who had experienced RM (range: 2–15), 14 of whom had living children, and three were pregnant at the time of interview^33 46^; see table 1. A minority of participants expressed a preference for a telephone interview because it was more convenient for them.

**Table 1:** Participant categories

Knowledge user group (identifier)	Number of participants
Clinical Midwife/Nurse Specialists in Bereavement and Loss (CMS)	8
Consultant Obstetricians/Gynaecologists (Hospital-based) (OBGYN-H)	4
Consultant Obstetrician (Private fertility sector) (OBGYN-FC)	1
Specialist Registrars (SPR)	2
Nurses (GN), Midwives (M), Sonographers (S)	4
Chaplaincy & Pastoral Care (CP)	2
Support Services (Perinatal Mental Health (PMH), Social Work (SW), Community & Voluntary (VS)	3
GPs (GP)	4
Practice Nurses (PN)	2
Public Health Nurses (PHN)	2
Administrative Support (AS)	1
Maternity Hospital/Unit/Group Level Administration, Governance & Management—Directors of Midwifery (DOM); Group Director of Midwifery (GDOM)	3
National Administration, Governance & Management (AGM)	6
Women who have experienced recurrent miscarriage, having carried the pregnancies (PW)*Four women experienced two first trimester miscarriages; nine experienced three or more*	13
Men who have experienced recurrent miscarriage (PM)*Two men experienced two first trimester miscarriages; five experienced three or more*	7
Total	62

Note: The 42 people involved in the management/delivery of services and supports were based at 29 different institutions or organisations at the time of interview. This included 14 of the 19 maternity hospitals/units in the Republic of the Republic of IrelandIreland. It should be noted that some had experience across a range of maternity hospitals/units, including as part of their training.

We generated one overarching theme from the data, which captures what RM services and supports should encompass based on participants’ accounts: ‘A standardised, dedicated, and adequately resourced and supported service in which women and men/partners experiencing RM are offered appropriate, individualised, timely and accessible care and support—this should begin following the first miscarriage and follow a graded approach’. Within this over-arching theme, there are three inter-linked themes: (1) dedicated staff, (2) dedicated space and time and (3) dedicated funding and support—prioritise RM (see table 2). Illustrative quotes are presented under each theme, with identifiers indicating the knowledge user group (see table 1) and participant number; for example, PW1 signifies a woman with lived experience of RM.

**Table 2:** Themes and subthemes

Overarching theme	A standardised, dedicated and adequately resourced and supported service in which women and men/partners with recurrent miscarriage are offered appropriate, individualised, timely and accessible care and support beginning following the first miscarriage and following a graded approach		
Themes	Dedicated staff	Dedicated space and time	Dedicated funding and support—prioritise recurrent miscarriage
Subthemes	Dedicated staff have specialist and experiential knowledge Staff need (ongoing) support and training Opportunity to strengthen primary care connections	Lack of dedicated or appropriate spaces The importance of giving time, yet time is constrained The need for non-judgemental, empathic, accessible and timely communication, information and support Recognising and meeting the needs of men/partners	Multilevel buy-in, collaboration, leadership and champions are needed to affect change in recurrent miscarriage care Evidence-based service change/delivery Funding and resource constraints Societal silence around miscarriage impacts on how it is perceived, prioritised (or not) and experienced

Much variation in practice was observed throughout participants’ accounts. Some maternity hospitals/units had dedicated RM clinics, while others did not, and people were seen by individual consultants within gynaecology clinics or private rooms and/or in private fertility clinics. It was also noted that even when a RM clinic existed within a hospital, private patients might not be referred into it by some consultants. Participants provided many examples of ‘disjointed’, ‘fragmented’, ‘haphazard’, ‘variable’ or ‘ad hoc’ care throughout the system—within and across hospitals (public/private) and other settings (eg, general practice, private fertility clinics)—with many people with lived experience ‘falling through the cracks’. The need for standardisation of RM services and care—including defined pathways and coordinated care—was evident. We will now explore themes regarding staff, space and time, and funding and resources which can contribute to service improvement efforts.

### Theme 1: Dedicated staff

*Dedicated staff* was a recurring theme across interviews. Participants described the need for dedicated staff in every sense of the word: having staff in dedicated RM roles and having staff that are dedicated in these roles, with adequate personal skills, training and supports. See table 3 for selected illustrative quotes; additional quotes are provided in online supplemental file 5.

**Table 3:** Illustrative quotes for Theme 1—Dedicated staff

Subtheme	Illustrative quote
1.1. Dedicated staff have specialist and experiential knowledge	‘… she (CMS-BL)was so kind and understanding towards me. She met me in the hospital a few times. She actually offered to come in to one scan with me after the missed miscarriage because she knew that I was terrified. She organised all my appointments, over the phone. Nothing was too much. And she always made sure that I was under *Consultant*, who knew me because after having *Daughter 1*… And they were very connected the two of them, in that like if there was questions or if there was issues like… Between *Consultant* and *Bereavement Midwife* I never had to worry about the extra things.’ (Woman with RM, PW9)
1.2. Staff need (ongoing) support and training	‘… I think it should be more a part maybe of training for NCHDs (Non Consultant Hospital Doctors) maybe. Like I said, what I have learned is literally only what I had to learn for exams and what I kind of learned through going through my years in different units. And someone else the same level as me who would have done that clinic would definitely have a lot more knowledge in the area. And it's only, like most people don’t get to do clinic, so I think we should have a bit more formal structured training in it, as part of our curriculum.’ (Consultant, OBGYN4)
1.3. Opportunity to better connect with primary care providers	‘I think they will absolutely need to be seen and managed in a secondary care setting…… And if I then were to receive you know a very comprehensive report, I can sit down with the couple afterwards and spend more time talking to them you know and going through it because of course we do that with everything, right. …… that’s why patients come to us as GPs all the time because we have that continuing care. We have that relationship with patients.’ (General Practitioner, GP2)

#### Subtheme 1.1: Dedicated staff have specialist and experiential knowledge

This subtheme describes the importance of having staff ‘dedicated’ to RM care; staff with the necessary skills, knowledge, expertise and attributes or qualities—including an interest and commitment to RM care. Having such staff in place also facilitates continuity of care and care coordination; something that people with lived experience valued—in particular not having to tell their story over and over, and the humanising, relational nature of care experienced. Health professionals—particularly Clinical Midwife Specialists in Bereavement and Loss (CMS-BL)—had ongoing relationships with people who had RM, from their initial losses through to subsequent pregnancies and beyond.

Participants described how ‘dedicated’ staff involved in the provision of RM care have specialist and experiential knowledge based on their training, professional development and clinical experience. Many working in RM and pregnancy loss have both a personal and professional interest in the area and drive service provision and development. Health professionals spoke about how they came into the area. Some were drawn to it by personal interest or lived experience (particularly CMS-BL), others were ‘landed’ in it (more often doctors). Health professionals spoke about the unknowns around RM, the dearth of evidence to support investigations and treatments and how they often relied on clinical experience and knowledge of individual patients to guide decision-making. Participants felt that in dedicated clinics, dedicated staff are seeing people with RM more regularly, which benefits care provision and experiences.

Most participants spoke about how CMS-BL are the linchpins of services, doing much visible and invisible work—providing bereavement care and signposting or referrals to further supports, care coordination and continuity of care, and staff education and training. Administrative staff/secretaries were also seen to provide important administrative and care coordination support.

When considering the importance of dedicated staff; however, a few mentioned how there can be gaps in provision when such staff members are on leave or change roles. Concerns were also voiced by a few health professionals about specialisation and how it can limit the availability of trained staff (particularly doctors) to run clinics.

#### Subtheme 1.2: Staff need (ongoing) support and training

Some health professionals noted how doctors-in-training can lack knowledge and experience around RM and bereavement care in general, with many relying on or benefitting from working with CMS-BL who guide them on care pathways and investigation and treatment protocols. This was also evident in the accounts of people with lived experience who—at length—provided examples of poor communication and care experiences with healthcare professionals. These encounters, which left them feeling frustrated, unheard, dismissed, distressed and disillusioned, reinforced the need for ‘dedicated’ staff.

CMS-BL also play a role in providing formal training for trainees, and indeed other hospital staff—including around care pathways and services (including referral criteria and processes) and bereavement care. While many noted that CMS-BL play a significant role in training, they acknowledged that they are stretched. Many health professionals highlighted that continuing professional development in RM is needed, with some noting that there can be a lack of interest or uptake from staff.

Some health professionals, and a few people with lived experience spoke about the impact of the role on health professionals or ‘vicarious trauma’. A few health professionals (primarily CMS-BL and sonographers) mentioned their own sources of emotional, psychological or professional support, primarily peer support—within or across hospitals, but also clinical supervision, self-care and the Health Service Executive Employee Assistance Programme. Participants noted that CMS-BL were a support to each other, but also to other staff within the hospital. Networking and collaboration among clinicians—particularly consultants and CMS-BL—was perceived as important to facilitate support, discussion of cases and service developments.

Some health professionals noted that the area can be unattractive and/or unrewarding due to the nature of RM, namely the relatively high level of unexplained cases and limited success of medical intervention beyond supportive care. That said, some mentioned how rewarding it was to care for and support women and couples with RM, particularly so when they went on to have ‘successful pregnancies’.

#### Subtheme 1.3: Opportunity to strengthen primary care connections

General practitioners (GPs) in particular were perceived as key contacts and sources of support for women and couples who experienced RM; yet communication and engagement with GPs around RM and miscarriage were viewed as haphazard. This included coordination of care between tertiary and primary care providers: ensuring GPs were informed of miscarriages, results of any investigations or treatments and plans for subsequent pregnancies.

While healthcare staff within RM or pregnancy loss clinics were seen to have specialist knowledge, primary care providers were perceived to lack sufficient knowledge in the area (often deferring to tertiary providers for guidance) but could be important sources of support for women. They often had established relationships and could provide continuity of care, including supportive care. It was also recognised that women who have a first trimester miscarriage may only engage with their GP, thus providing an important point of contact.

### Theme 2: Dedicated space and time

Dedicated space and time focus on both the physical and relational space needed for RM care; relational here describing both the spatial and temporal aspects of care. Key issues within this theme are inappropriate physical spaces—those shared with people who have different pregnancy experiences, the importance of time and timely, accessible care and the need to create space for men/partners within RM services and supports. See table 4 for illustrative quotes; further quotes are provided in online supplemental file 6.

**Table 4:** Illustrative quotes for Theme 2—Dedicated space and time

Subtheme	Illustrative quote
2.1. Lack of dedicated or appropriate spaces	‘And even though I know we do scan those women in the mornings only that’s kind of the best we have. But they’re just consumed within the whole hospital setting you know so they’re part of that. And I suspect that they may feel they don’t matter, which is not what you’re trying to do. But I suppose the logistics of trying to provide a separate service would probably be helpful you know.’ (Clinical Midwife Specialist in Bereavement and Loss, BM2)
2.2. The importance of giving time, yet time is constrained	‘We met *Consultant*, and she talked us through… and sure I was in such a state. And she was very good, and *Bereavement Midwife* explained it again. And I thought she was going and then she’d turn around and she’d say to me do you understand it and I’d say no. I didn’t get it. So she sat down and she started again. And I’m sure you know I often wonder did they put in an extra bit of an effort because with any other clinic appointment you wouldn’t get it. I will say that for them. She sat down and she would have said it all again and gave us time to ask questions. You know I’m sure she was on a time scale and there was a rush, but they did give hugely when I got in there.’ (Woman with RM, PW2)
2.3. The need for non-judgemental, empathic, accessible and timely communication, information and support	‘… I’m not going to slate doctors because I’ve met, you know, lovely, lovely doctors along the way, but my God the nurses pick up after them all the time it feels…… There was other times I’ve gone in, you know, after about ten miscarriages… I went in to get a scan done to confirm that it was the miscarriage, and the doctor turned around and said, ‘well, you’re young and, you know, it’s probably just because of a genetic abnormality and you can try again, and I’m sure the next time everything will be alright’. And I just looked at him and I said, ‘have you even looked at my file?’… And he just looked at me. And I just got up out of the room and I walked.’ (Woman with RM, PW11)
2.4. Recognising and meeting the needs of men/partners	‘I think they could do with a section in the booklet (from the hospital) about how for a partner to deal with it as well, or even just tips on how to provide support. Because I think you come out of it just feeling like you have your own loss, and you have the loss of the two of you together, but you also realise like you have to provide some sort of emotional support. And it’s one of those like you can’t see the wood for the trees kind of moments because you’re like which do I deal with, do I deal with their emotions, do I deal with mine, is there a way to deal with them together? But I think there’s no real advice that’s given out on that.’ (Man with RM, PM5)

#### Subtheme 2.1: Lack of dedicated or appropriate spaces

Participants spoke, at length, about the lack of dedicated space for RM/pregnancy loss care within maternity units and hospitals. Waiting areas and spaces where ‘bad news’ was communicated were of particular concern. This had an extremely negative impact on women and couples, who also stressed the lack of compassion and humanity demonstrated by inadequate physical spaces. Many mentioned how this was often the source of complaints and, in some places, advocacy efforts by staff and patient representatives.

Some health professionals spoke about efforts they made to minimise the impact of limited or inappropriate physical spaces on women and couples, including preparing them for what to expect in advance, and advising them of alternative routes to the clinic to avoid areas that could be triggering. Staff also spoke about improvements that had been made to physical spaces within hospitals arising from philanthropic efforts or health service funding. Despite such efforts, staff voiced concerns about their limitations due to larger infrastructural issues (eg, old buildings). For many participants, appropriate spaces to ensure compassion, dignity, respect and privacy were a key area for service improvement.

#### Subtheme 2.2: the importance of giving time, yet time is constrained

The importance of giving time to women and men who experience RM was highlighted throughout participants’ accounts. This included giving women/couples time at the point of loss and/or at reassurance scans in subsequent pregnancies and also at clinic appointments—to ask questions, take detailed histories, provide information and explanations about RM and their care, emotional support and reassurance, or just to be physically with them as they processed events or information communicated to them. For many people with lived experience, however, the time afforded to them varied within and across services.

Many health professionals spoke about how it was difficult to provide a good service due to high patient numbers, with staff doing their best under the constraints in which they operated. Some stated that clinics were run at a particular frequency to match resources such as staff and space, as well as allowing sufficient time for consultations; however, this could mean longer waiting times for appointments.

For health professionals, this often meant not having time to provide supportive care as this was just one of several types of pregnancy loss that came under their care. This was particularly relevant to CMS-BL who felt it compromised their identity as providers of bereavement support. Some participants also felt that this lack of time compromised patient care and was difficult for staff. Emergency care was seen to be the priority, particularly high-risk cases, with lower priority assigned to miscarriage and/or bereavement care within services and beyond. Demands on time could impact on how care is provided or experienced, with many women and men with RM noting the time pressures that health professionals were under.

#### Subtheme 2.3: The need for non-judgemental, empathic, accessible and timely communication, information and support

The need for non-judgemental, empathic communication—from the first miscarriage experienced through any subsequent pregnancies—was highlighted. This included during communications about risk factors (particularly regarding age) and advice regarding behavioural and/or other changes that participants could make to prepare for subsequent pregnancies. Health professionals often discussed this in the context of trying to ‘empower’ women/men, when they could often feel powerless given the nature of RM. The need for respectful, supportive and empathic communication with health professionals around the time of loss, including ‘breaking bad news’ was also highlighted, with many people with lived experience recounting negative experiences and impacts.

Timely communication and information are also needed. This includes providing women/couples with information about miscarriage/RM in advance—to prepare them for what might happen around the time of loss, decisions around miscarriage management, RM/pregnancy loss clinic appointments and/or to set their expectations of pregnancy and how one in four pregnancies can end in miscarriage. Health professionals noted the importance of informing people that certain aspects of care can take time (eg, confirming a miscarriage, RM investigations), keeping them updated and ensuring that they are sign-posted to supports. This was a key issue for people with lived experience, particularly women, who spoke at length about lack of follow-up and how they had to constantly chase services/staff for information.

Many participants highlighted the need for written information regarding RM and more up-to-date, relevant information resources. Some noted that people (or they themselves) may seek information/support online—and elsewhere—if they were not engaged with hospital services and/or felt that these were lacking; prompting them to do their own research. The need for information resources in languages other than English, and timely and appropriate interpreter services was mentioned by a few health professionals also.

The importance of having access to supports, when they needed them, was mentioned by many with lived experience, including greater access to early pregnancy units for scans and CMS-BL. In some instances, women with lived experience spoke about making contact with supports (eg, CMS-BL), but when they did not receive a response, this deterred them from doing so again.

People with lived experience stressed the need for timely communication of results and an opportunity to discuss these and plans for any future pregnancies—with many stating that this did not happen in their case. Similarly, many felt that health professionals could be more proactive in terms of investigations and treatments they offered, with many feeling that they had to ‘push’ for these based on their own research. Health professionals discussed ‘going by evidence’ but also needing to do something—feeling constrained by current evidence, some provided individualised investigations and/or treatments (or not) based on their clinical experience and expertise. They spoke about the importance of giving time within clinics to explain such matters and manage expectations. People with lived experience echoed this and appreciated—or would—when health professionals acknowledged that they might not have all of the answers, or the limitations of current evidence.

#### Subtheme 2.4: Recognising and meeting the needs of men/partners

Participants spoke about how the needs of partners were under-recognised and unmet within services, highlighting the importance of dedicated space and time to address these needs. Many spoke about how men/partners reacted differently to miscarriage and RM, prioritising the views, feelings and needs of their partners while sidelining and struggling to make sense of their own grief and emotions due to social and cultural norms of masculinity. Men/partners perceived their role as one of a supporter and protector, feeling that they could not legitimately play any other role as they did not physically experience the pregnancy or its ending. They felt unprepared for, shocked and fearful when confronted with the physical realities of miscarriage. There was varying involvement of men/partners in hospital or GP appointments, often dependent on whether they could get time off work or not, or indeed if their partner wanted them, or felt they needed to be there. They tended not to seek out or avail of bereavement support, either through the hospital or community.

Women and men with lived experience spoke about the impact of RM on their relationships. Men often wanted to stop particular investigations, treatments or even trying to conceive again to ease the pain and suffering of their partner, who wished to persist, which could fracture relationships. Many health professionals spoke about actively encouraging men/partners to attend appointments and the importance of acknowledging their loss and grief. The need for dedicated information for men/partners was highlighted, as well as ways to better involve, engage and support them in RM care.

### Theme 3: Dedicated funding and support—prioritise RM

The third and final theme relates to broader system issues which influence how RM, and indeed pregnancy loss more broadly, is perceived, understood, (de)prioritised and how these impact—or could be harnessed to positively impact—policy and practice. See table 5 for illustrative quotes; further quotes available in online supplemental file 7.

**Table 5:** Illustrative quotes for Theme 3—Dedicated funding and support—prioritise recurrent miscarriage

Subtheme	Illustrative quote
3.1. Multilevel buy-in, collaboration, leadership and champions needed to affect change in recurrent miscarriage care	‘Like some hospitals have a lead clinician for pregnancy loss services but not every hospital has that. So where you have a hospital, where you have a consultant who has a responsibility or an interest, they’ll drive it with the bereavement midwives. In other places, it depends…… It depends on the culture around bereavement care I suppose, and the level of importance that it has within that particular organisation.’ (Person involved in National Administration, Governance & Management, AGM1)
3.2. Evidence-based service change/delivery	‘I think there is a very strong case for setting standards and setting certain core objectives that should be applicable in all nineteen units and indeed in the private sector as well. And I think if it was standardised in the nineteen and put it out there, for not just the professionals, put it out there for the women themselves that this is how its defined, this is what you should expect and if somebody is offering you certain treatments well then you should ask them for the evidence that these treatments work. Because I mean that really gets me upset when I hear people getting experimental treatments that haven’t been shown to be of benefit.’ (Person involved in National Administration, Governance & Management, AGM5)
3.3. Funding and resource constraints	‘But again, I mean *Bereavement Midwife* is not a counsellor. You know they can only do so much. Whereas they don’t seem to have access to the services that are actually required maybe if they listened to what women were saying that they might actually be able to put something effective in place. Even simple things like I can’t understand how the early pregnancy clinic is open Monday to Friday 9 till lunchtime. I mean it just beggars belief. Women have miscarriages all the time… And I think that’s the other thing with this is that you’re not a priority when you’re having a miscarriage because the pregnant women are a priority. You’re not. You don’t feel that care and attention. You just don’t get it because like you’re not pregnant. So it’s almost like well your baby is dead so you know we don’t really need to deal with you but we deal with the people who are actually having babies.’ (Woman with RM, PW1)
3.4. Societal silence around miscarriage impacts on how it is perceived, prioritised (or not) and experienced	‘And even on my first pregnancy I suppose, of course when you’re pregnant and things seem to be going well, like of course they’re not going to say, ‘by the way, you know there’s a really high chance you could still have a miscarriage in the first twelve weeks’. And I just feel like I don’t know whether it would be good if doctors or GPs would say that, just to kind of normalise it in some way, or just so you’ve heard it really once in your life.’ (Woman with RM, PW13)

#### Subtheme 3.1: Multilevel buy-in, collaboration, leadership and champions needed to affect change in RM care

Health professionals noted that support and advocacy were needed to affect change in RM care at national and local level (including group/hospital-level); the latter in particular accounting for what works best relative to their context. This required having buy-in from a wide range of people or groups such as the National Women and Infants Health Programme in the Health Service Executive, the Royal College of Physicians of Ireland, hospital groups, hospitals—including clinical directors, directors of midwifery, consultants, CMS-BL and support groups.

Many—particularly doctors and CMS-BL—stressed how individuals with an interest in the area could drive (and often persist in the face of adversity with) service delivery or improvement efforts, regarding dedicated spaces for RM or pregnancy loss, education programmes or referral systems, among others. While some services had such champions, predominantly in the form of individual consultants/CMS-BL, others did not. Participants also felt that the people involved needed to be ‘the right’ people, with the appropriate insights or expertise, ‘not just a job title’. Some mentioned how patient advocacy informed or drove service improvements, primarily locally, but also nationally.

Those who championed service improvements spoke about resistance to change encountered, with some stating that things had to ‘go badly wrong’ before change is initiated. RM was perceived as low down in terms of healthcare priorities and funding, or investment in RM unjustified due to the low numbers within some services.

Power relations (and sometimes politics) permeated participants’ narratives. For example, health professionals could facilitate or ‘deny’ people access to RM care (eg, based on referral criteria)—though ultimately the ‘lead clinician’ decided how RM services would be delivered and to whom; clinicians (and others) could also suppress, or sideline knowledge arising from people’s lived experience of RM and produce knowledge through their own professional practice. People in management/governance roles within hospitals, hospital groups or at national level could prioritise funding and resources for RM care, or not. People could exercise discursive power in how they spoke, or not, about pregnancy loss and the language used. The nature of RM and the limited evidence for investigations and treatments also wielded power over all within the system. A supportive organisational culture was deemed necessary to advocate for and provide good quality RM, pregnancy loss and bereavement care. Together it was felt that, through multilevel buy-in, collaboration, leadership and champions of multilevel collaboration, people could use their power to affect meaningful change.

#### Subtheme 3.2: Evidence-based service change/delivery

Health professionals and people with lived experience noted variation in practices and structures within and across maternity units/hospitals and the impact that this had on care experiences. As noted previously, the need for standardisation of RM services to ensure a standard (and equitable) level of access, provision and care was evident. The predominant view was that only evidence-based investigations and treatments for RM should be offered; however, many also offered empiric treatments ‘to do/try something’ without the potential for harm, in consultation with the individual/couple involved, noting the limitations in supporting evidence.

Participants—health professionals and people with lived experience of RM—spoke about many unconventional investigations and treatments, often available within the private sector, and the potential for harm. While people with lived experience were often aware of the limited evidence and potential associated harms, they sometimes pursued these to try everything they could to not have another miscarriage—feeling that the public system could have done more for them. It was clear that, for many, treatments for RM were often on a trial-and-error basis, seeing if something worked or not, and then trying something else; piecing the puzzle together as they went along, which is indicative of the nature of RM. People with lived experience generally valued such approaches, as well as explanations received.

Many health professionals spoke about how service improvements or changes should be evidence-informed; this was needed to make a convincing argument for change within services and systems. The lack of available data on numbers of people who experience miscarriage/RM was highlighted by some health professionals as a barrier to service planning and improvement efforts.

#### Subtheme 3.3: Funding and resource constraints

Lack of funding was a widely cited constraint to delivering the desired level of RM care. It was often discussed in terms of advocacy or proposals put forward to management for service improvements, or as a reason for having strict referral criteria for RM (often three consecutive miscarriages). It was also discussed in the context of genetic testing—with many speaking about how they would like to have greater genetic testing of pregnancy tissue and access to genetic counsellors. Lack of access to or availability of psychological supports was another key issue, particularly around the time of loss and in subsequent pregnancies. Some people with lived experience spoke about how CMS-BLs could ‘only do so much’ and that access to further supports within hospitals (eg, psychology, social work) were limited, which meant that people had to pay privately to access them.

#### Subtheme 3.4: Societal silence around miscarriage impacts on how it is perceived, prioritised (or not) and experienced

Many participants highlighted how the silence around miscarriage/pregnancy loss impacted on how it was perceived—by all—influencing public understanding and expectations of people with lived experience, as well as prioritisation by health service decision-makers; ultimately impacting the prioritisation, resourcing and delivery of services and how women and men experienced pregnancy loss and subsequent care and supports.

The silence was perceived to be influenced by the cultural and historical factors in the Republic of Ireland regarding the treatment of women and pregnancy endings, the associated stigma, shame and guilt, and within that the normalisation (and indeed minimisation) of pregnancy loss. This was felt particularly relevant to first trimester pregnancy loss, which could be perceived as less significant than later losses.

Participants noted how people do not talk about miscarriage/pregnancy loss, and often only think or talk about it when they experience it, or meet someone who works in the area. That said, some women and men spoke about how they now openly talked about their losses, for example, in the workplace. While increasing media attention in recent years was generally welcomed, some felt that more consistency and evidence-informed education or messaging was needed, as well as diversity in voices or experiences. For example, hearing from people who do not have a ‘live’ baby following RM, and/or who decide to stop ‘trying’ despite all of the efforts and investments on their part.

Many spoke about people’s expectations of pregnancy—and the perception that getting pregnant and having a baby would be relatively straightforward. People with lived experience felt unprepared for pregnancy loss, never mind recurring losses. For some women, despite being aware of miscarriage, they did not think it would happen to them. Participants highlighted the need for greater education and awareness campaigns, including within schools and communities. A few participants highlighted the potential to increase anxiety with public awareness/information campaigns and the need to avoid or minimise this.

## Discussion

In this study, we aimed to better understand RM practices and experiences, and factors influencing the provision of desired services and supports, through interviews with service providers and people with lived experience. The three themes generated emphasise the need for dedicated staff, space and time, and funding and resources.

Our analysis provides support for a graded approach to RM care.^32^ People’s experiences and perspectives were shaped from their first miscarriage; better meeting their needs at this point, by offering healthcare advice and support, may improve experiences and outcomes. Care can then be augmented following two miscarriages and three miscarriages, via nurse or midwife-led clinics and medical consultant-led clinics, respectively.^32^ Poor healthcare support experiences can exacerbate adverse psychosocial outcomes following miscarriage.^2 47^ Service provision was experienced, and sometimes delivered, in a very disjointed way with much opportunity for improvement.

Evident from our analysis are the affective, cognitive, temporal and material demands of RM for people with lived experience as they negotiate both the direct impacts of successive miscarriages and navigating the healthcare system. Women experienced a substantial ‘burden of responsibility’ in their care—pursuing investigations, results, treatments, information and supports, often feeling dismissed, disrespected and deprioritised; similar has been reported in other areas of healthcare.^48^ Even when appropriately investigated, approximately half of RM cases can be unexplained.^49^ People with lived experience appreciated honesty from healthcare professionals when they reached the boundaries of available evidence or their own knowledge.^48^

Our findings align with current RM clinical guidance from the European Society of Human Reproduction and Embryology^49^ and Royal College of Obstetricians and Gynaecologists^50^ (though how they define RM varies) regarding the importance of appropriate investigations and treatments, dedicated clinics, psychological supports, and supportive care in subsequent pregnancies. Dedicated RM clinics can provide necessary care, supports and continuity,^51^ and people who attend dedicated RM clinics generally have a relatively high live birth rate in a subsequent pregnancy; in the Republic of Ireland, 63%–75%.^52 53^ In our study, access to dedicated clinics varied, not only in terms of not meeting referral criteria,^33^ but availability of clinics nationally also.^54^

### Dedicated staff

Broadly, people want dedicated and appropriately trained staff involved in RM care, including a lead clinician for RM and CMS-BL within clinics. The central role of the CMS-BL was evident and has previously been noted in relation to miscarriage care, particularly regarding emotional support.^13^ Though only raised by a few health professionals, the perceived negative impacts of specialisation in RM need to be addressed and basic training for all provided; this should include education around RM clinical guidelines. Lack of knowledge and training (particularly communication skills) has previously been reported by midwives/nurses^29 55 56^ and doctors-in-training.^57^ Similar to research on the impact of caring for people who experience later pregnancy losses,^28^ people involved in providing RM care require institutional acknowledgement, education, training and supports to enhance psychological well-being and bereavement care. Unsupportive organisational cultures (including constrained time and spaces) inhibit the provision of good pregnancy loss care.^29^ It can also contribute to moral distress^58^ as evidenced in health professionals’ accounts in our study. The emotional demands of caring have been identified by nurses and midwives in the care of involuntary pregnancy losses.^29^ Furthermore, people who frequently care for women experiencing miscarriage are at risk of becoming indifferent to the personal significance of miscarriage because of frequent exposure, or can withhold providing extensive emotional support as a form of self-protection.^30^

As other studies have shown, support offered by GPs to people with RM can vary; while they can be the first health professionals that people with RM access for support in a subsequent pregnancy, lack of support and understanding can be experienced.^19^ The role of primary care in the care of people with RM warrants further consideration, with opportunities for education and training and more joined-up approaches to investigations, treatments and supportive care with hospitals/RM clinics.

### Dedicated space and time

Inadequate physical spaces within hospitals—including emergency departments and general clinics—are frequently cited concerns of people who experience miscarriage/pregnancy loss and can amplify negative emotions^10 13 59^; our findings are no different. While efforts were made to counter these issues by staff, people with lived experience still felt the negative impacts.

As observed in other studies involving people with experience of miscarriage/RM,^10 22 47 56 59^ people with RM reported mixed experiences with healthcare providers, with almost all identifying areas where support and sensitivity were lacking. Lack of information, follow-up care, acknowledgement of the psychological impact of RM, emotional support or sign-posting/referral to supports around the time of miscarriage/RM have been highlighted.^4 9 10 22^ Women want healthcare providers to be proactive in offering support, information and emotionally sensitive care at the time of miscarriage.^47 56^ Distress can be exacerbated otherwise.^10^ Earlier access to treatment, support and follow-up can help people to cope with the negative impacts of RM.^4^ Supportive care—including reassurance scans and emotional support/counselling—and continuity of care in subsequent pregnancies are important for people with RM, as noted in this study and elsewhere.^13 19 20 24 51^ Insufficient time and resources have previously been reported as barriers to supporting women experiencing miscarriage.^30^ Improvements could be made by providing information and explanations, discharge and follow-up instructions, and through discussions about available supports,^17 59^ as well as follow-up phone calls after a miscarriage.^17^ Given the constraints to providing supportive care, innovative approaches are needed.^60^

Men/partners also need to be better engaged by RM services. As noted in other studies, men feel that their main role is to support their partner, that they need to suppress their own feelings and grief, and that they are overlooked and marginalised by services.^4 14 16 18 21 23^ Greater appreciation of the impacts of RM on men/partners is needed, as well as accessible supports, tailored to their needs and preferences.^20^

### Dedicated funding and resources—prioritise RM

Addressing broader, systemic issues is necessary to enhance RM services and supports. In our work, people felt that buy-in, collaboration and leadership at all levels of the system were required, as well as evidence to support service improvements. Funding and resource constraints are continuing challenges to delivering the desired level of services, supports and care. Financial investments are needed to address some of the issues identified within this study.

Lack of knowledge and awareness of miscarriage, and indeed how this can be shaped by societal silence and stigma, remains an issue.^13 61 62^ The silence surrounding miscarriage can lead to feelings of loneliness and isolation and poor support experiences.^8^ As evidenced in our study, first trimester loss can be perceived as further down on the ‘hierarchy of loss’^63^; people often disregard the potential impact of such losses, even if recurrent, including the ‘loss of possibility’.^61^ Ending the silence, and better public understanding, is vital for people to process their grief/loss, access supports and navigate everyday communications.^51 64^ Some would argue, however, that concerns about the public silence surrounding miscarriage are overstated and that instead more emphasis needs to be placed on addressing structural inequalities.^65^

Participants in our study felt that more education in schools—including appropriate communication strategies—was needed, reinforcing previous calls of this nature.^62 66 67^ Education on evidence-based investigations and treatments for RM is also needed, for health professionals and people with lived experience alike. The offering and use of unconventional investigations and treatments, particularly within the private sector, must be addressed. People with lived experience clearly valued explanations from health professionals; therefore, providing information and support even within the uncertainty will help. Dedicating appropriate space and time within clinics—and more broadly—to communicating the unique challenges that RM presents, the limited evidence-based investigations and treatments available, and prognosis for future pregnancies may assist in managing expectations and informed decision-making. Investing in, and standardising and structuring, RM care within the health service in line with clinical guidelines and a graded model may alleviate people’s perceptions that the public system could have done more for them.

Issues raised within this study—including poor communication, inadequate physical spaces and stigma—are long-standing issues within miscarriage research, enduring over four decades.^68^ There appears to be systemic failure globally to address such issues. Women’s healthcare is often invisible or not prioritised by political parties in the Republic of Ireland^69^ and elsewhere.^70^ We share calls by others for Governments to prioritise and (sustainably) resource practice and research in women’s health issues,^71^ including miscarriage and RM.^72^ Involving knowledge users (including people with lived experience, health professionals and others) in service improvements from project conception through to the implementation of arising innovations (eg, guidelines, toolkits, models or services) is vital, and can enhance the sustainability of innovations.^73^ Globally, efforts to prioritise and invest in miscarriage care are beginning^74 75^; this study provides further insights into what is needed at various levels of our systems to enhance RM services and supports.

### Strengths and limitations

The inclusion of a diverse range of perspectives—including people with lived and professional expertise—is a strength of this study. We did not limit our gaze inwards on services; we also situated our evaluation within broader healthcare, social and political contexts. It enabled us to access rich insights and contextual understanding into systems of RM care that might not have been garnered otherwise. While our study was conducted during the early phases of the COVID-19 pandemic, our analysis focuses on broader experiences; we have reported on experiences relating to the pandemic separately.^46^ A limitation of our study, similar to other studies of the lived experience of RM,^51^ is the lack of ethnic/cultural diversity within the sample. For example, our analysis may underestimate the need for interpreters and multilingual resources, among other issues. Research is also needed in this area on the needs of people who do not identify as cis-gender women. While our data were generated between June 2020 and February 2021, the analysis remains relevant as little has changed in policy and practice in the Republic of Ireland during this time. An RM service evaluation conducted in 2021/2022 noted similar findings.^54^ Despite the introduction of a clinical practice guideline for RM care in 2023,^76^ there has been little investment in RM, or pregnancy loss more broadly—an issue of relevance to other countries also.^72^

## Conclusions

Dedicated staff, dedicated space and time, and dedicated funding and resources can contribute to enhancing experiences of RM and RM care, service provision, and better healthcare outcomes. An adequately resourced, graded approach to miscarriage care is needed in tandem with clinical guidelines and defined care pathways. This requires actions at multiple levels within the system, including: prioritising miscarriage and RM care, adequately funding and resourcing services, enhancing health professional education and support, greater care coordination within and between hospitals and primary care, and improving public awareness of, and addressing stigma surrounding miscarriage. In light of recent debates regarding how best to deliver RM care, and changing international guidelines, this work provides timely and important knowledge that should be harnessed to inform service improvement efforts in the Republic of Ireland and beyond.

## Supplementary material

10.1136/bmjopen-2024-094753 online supplemental file 1

10.1136/bmjopen-2024-094753 online supplemental file 2

10.1136/bmjopen-2024-094753 online supplemental file 3

10.1136/bmjopen-2024-094753 online supplemental file 4

10.1136/bmjopen-2024-094753 online supplemental file 5

10.1136/bmjopen-2024-094753 online supplemental file 6

10.1136/bmjopen-2024-094753 online supplemental file 7

## Data Availability

No data are available.
