# Supplementary material for: Views of knowledge users on recurrent miscarriage services and supports in the Republic of Ireland: a qualitative interview study
Source: BMJ Open. 2025 Apr 10;15(4):e094753. doi: 10.1136/bmjopen-2024-094753 (PMC11987160; doi:10.1136/bmjopen-2024-094753)
Supplement: online supplemental file 1 [file bmjopen-15-4-s001.docx]

**Supplementary File 1: Additional information on the study context**

The RE:CURRENT (REcurrent miscarriage: evaluating CURRENT services) project^1^ was conceptualised in 2018/2019 and commenced in January 2020 further to receipt of funding from the Health Research Board. Interviews for this qualitative study were conducted from June 2020 to February 2021. At that time, there was no national clinical guideline for recurrent miscarriage. Recurrent miscarriage was defined as three consecutive miscarriages in the Republic of Ireland, though internationally definitions were being revised in places to encompass two miscarriages, whether consecutive or not.^2^

In the preceding decade, several events laid the foundations for change in how miscarriage services and care were delivered in the Republic of Ireland. Further to cases of misdiagnosis of miscarriage in 2010, and a resultant audit commissioned by the Health Service Executive (HSE), recommendations made by the Miscarriage Misdiagnosis Review Team were implemented by the HSE. These included the establishment and enhancement of Early Pregnancy Assessment Units, the development of ultrasound training programmes, and the provision of new ultrasound equipment and software.^3,4^ A national clinical guideline on miscarriage were developed and distributed to all hospitals,^4,5^ but there was no guideline – or indeed recommendations – specific to recurrent miscarriage. Around this time also, reviews into perinatal deaths in the Republic of Ireland recommended bereavement care after pregnancy loss as an integral part of maternity services. Thus, the HSE published and implemented the National Standards for Bereavement Care Following Pregnancy Loss and Perinatal Death.^6^ As part of this work, pregnancy loss services were reviewed in all 19 maternity units/hospitals in the Republic of Ireland. At the time of the review (2016), only four units had dedicated recurrent miscarriage clinics.

A national evaluation of recurrent miscarriage services in the Republic of Ireland – conducted as part of the RE:CURRENT Project between November 2021 and February 2022 – found considerable variation in practice.^7^ This included variation in referral criteria and processes; information provision; clinic location (close to antenatal clinics, wards or other areas where pregnant women may be seen, or not); access to psychological supports, laboratory facilities, genetic counselling, 3D ultrasound; investigations and treatments undertaken outside of recommendations; and recording of subsequent pregnancy-related outcomes. Nine services (50%) stated that they had a dedicated recurrent miscarriage clinic, while nine did not – in these services women were seen in a gynaecology clinic (n=7), early pregnancy unit (n=1), or reproductive clinic (n=1).

In January 2023, the first national clinical guideline for recurrent miscarriage was published; this redefined recurrent miscarriage as the loss of two consecutive miscarriages in the first trimester.^8^

In the Republic of Ireland, the Department of Health is responsible for developing policy, and the Health Service Executive is responsible for implementing policies and health service provision. The Republic of Ireland’s first national maternity strategy was published in 2016;^9^ however, there was little mention of miscarriage, and indeed recurrent miscarriage, within this strategy. Reference was made to the need for: women who experience miscarriage or receive bad news to be cared for in appropriate spaces separate to those for pregnant women or newborns; psychological support for couples who experience pregnancy loss (in a hospital or primary care setting); offering a follow-up appointment to women with recurrent miscarriage for further investigations – but these were not formal recommendations or actionable items within the strategy. The National Women and Infants Health Programme within the HSE, established in 2017, is responsible for the implementation of the national maternity strategy, and the management, organisation and delivery of maternity, gynaecology and neonatal services in Republic of Ireland. It is also responsible for the aforementioned National Standards for Bereavement Care Following Pregnancy Loss and Perinatal Death, which were updated in 2022.^6,10^

Healthcare – and indeed maternity care – in the Republic of Ireland is provided on a public and/or private basis. All maternity services within the publicly funded healthcare system (general practice and maternity unit/hospital) are provided free of charge.^11^ People may opt for private maternity care if they so wish, and have the financial means to do so.

**References**

1 Pregnancy Loss Research Group. RE:CURRENT. University College Cork. 2024. https://www.ucc.ie/en/pregnancyloss/researchprojects/recurrent/ (accessed 29 June 2024)

2 Hennessy M, Dennehy R, Meaney S, *et al.* Clinical practice guidelines for recurrent miscarriage in high-income countries: a systematic review. *Reproductive BioMedicine Online*. 2021;42:1146–71. doi: 10.1016/j.rbmo.2021.02.014

3 Health Service Executive. National Miscarriage Misdiagnosis Review. Dublin: Health Service Executive 2011.

4 Ledger WL, Turner MJ. Implementation of the findings of a national enquiry into the misdiagnosis of miscarriage in the Republic of Ireland: impact on quality of clinical care. *Fertility and Sterility*. 2016;105:417–22. doi: 10.1016/j.fertnstert.2015.11.002

5 Royal College of Physicians of Ireland. Management of Early Pregnancy Miscarriage. Dublin: Royal College of Physicians of Ireland 2012.

6 Health Service Executive. National Standards for Bereavement Care following Pregnancy Loss and Perinatal Death. Dublin: Health Service Executive 2016.

7 Hennessy M, Linehan L, Flannery C, *et al.* A national evaluation of recurrent miscarriage care services. *Irish Medical Journal*. 2023;116:P713.

8 Linehan L, Hennessy M, Khalid A, *et al.* National Clinical Practice Guideline: Recurrent Miscarriage. Dublin: National Women and Infants Health Programme and The Institute of Obstetricians and Gynaecologists 2023.

9 Department of Health. Creating a Better Future Together: National Maternity Strategy 2016-2026. Dublin: Government Publications 2016.

10 Health Service Executive. National Standards for Bereavement Care following Pregnancy Loss and Perinatal Death. Version 2. Dublin: Health Service Executive 2022.

11 Citizens Information. Maternity and Infant Care Services. 2022. https://www.citizensinformation.ie/en/health/health-services/reproductive_health/maternity-and-infant-welfare-services/ (accessed 3 July 2024)
