# Supplementary material for: Views of knowledge users on recurrent miscarriage services and supports in the Republic of Ireland: a qualitative interview study
Source: BMJ Open. 2025 Apr 10;15(4):e094753. doi: 10.1136/bmjopen-2024-094753 (PMC11987160; doi:10.1136/bmjopen-2024-094753)
Supplement: online supplemental file 2 [file bmjopen-15-4-s002.docx]

**Supplementary File 2: Topic Guide – People with lived experience**

*Suggested probes are outlined below. These should be utilised where appropriate and only if needed to promote discussion. You do not need to ask all questions, be guided by the participant*

**Background/Contextual Information (can be collected in course of interview if more appropriate)**

- Can you tell me a little bit about yourself-age/relationship status/children/profession
- Can you tell me about the number of losses that you have experienced and when? Number of losses/order of losses/weeks gestation

**Expectations and Experience of Pregnancy before Loss**

- What were your expectations for pregnancy before miscarriage?
- Can you tell me about your experience of pregnancy before miscarriage?

**Experience and Management of Recurrent Miscarriage (two or more miscarriages)**

- What were your expectations of care after experiencing multiple miscarriages (two or more)?
- Did you enquire about investigating the miscarriages at any point or did anyone talk to you about investigating the miscarriages? (After how many losses was this discussed/what were you advised/management of miscarriage-conservative, medical, surgical)
- *If offered investigations and follow-up*-Can you take me through the care you received and how this came about?
  - *Did you opt for public or private care*? Why?
  - Did you get a referral, from who, to where and for what? How was this communicated?
  - What were you asked to do?
  - What did you hope to get out of the appointment?
  - How long were you waiting for appointments?
  - Where did you have to go? (phlebotomy clinic, gynae ward, etc.)
  - Did anyone go with you?
  - Who did you meet? (consultant, midwife, SPR, nurse)
  - What was discussed? Did you receive results? Was treatment or a plan of care discussed? How did you feel about this?
  - How did you feel about the appointment and any plan that was discussed? (ask about space/environment if not mentioned)
  - Did anyone speak to you about future pregnancies? (Who/what information/advice was provided/were you satisfied with the information/advice)?
- *If not offered/refused investigations and follow-up-*Can you tell me about the reasoning that was given-if any, how did you feel about this? Were you given any other advice at this point?
- Did you at any point consider or seek a second opinion (or private care)? If so, who and why? Can you tell me about this?

**Subsequent Pregnancy**

- Can you tell me about your experience of pregnancy after a previous loss? What were your expectations? How did you feel?
- Can you tell me about the care received in your pregnancies after loss? (GP/hospital/public or private care/Reassurance scan/clinic)
- If a treatment plan was put in place for this pregnancy, did you know what to do, where to go?
- Were you offered or did you avail of any support in subsequent pregnancy(ies)? (formal/informal)

**Knowledge and Understanding**

- Before experiencing miscarriage, did you feel like you knew enough about pregnancy, fertility, pregnancy loss? Where did you get this information? What would you liked to have known? Would you do anything different, given your experience / what do you know now?
- Did you feel like you were informed enough about the care you received for miscarriage/recurrent miscarriage? (investigations, treatment, subsequent pregnancy plan)
- What kind of information was provided (what format) and was this useful? -were these useful? What would you liked to have known?
- Did you seek information elsewhere? (professional/family/friends/support organisations/internet)? Did you speak to any of your care providers about this?

**Impact**

- In your experience, what has been the impact of recurrent miscarriage on you and your family? (self/partner/children/wider family-work, social, mental and physical health/financial)

**Support Received or Sought**

- Did you feel that you needed support at that time?
- Can you tell me about any support offered to you or your partner?
- Did you avail of any supports? (hospital/GP/private counselling/support organisation/family/friends). How did you hear about these supports and what encouraged you to attend? What happened? Did this meet your needs at the time?

**Experience/Recommendations**

- Reflecting on the care you have received for recurrent miscarriage-what are you overall thoughts about your experience?
- What do you think have been the positives-what worked well?
- What are the negatives, is there anything that did not work so well?
- What would you like to have happened?
- Is there anything that health professionals could/should have done differently?
- Is there anything that you would do differently?
- Do you have any recommendations to improve the care and support for those who experience recurrent miscarriage?

**Is there anything that we haven’t touched on that you feel is important to mention? / Do you have anything else to add?**
