# Supplementary material for: Views of knowledge users on recurrent miscarriage services and supports in the Republic of Ireland: a qualitative interview study
Source: BMJ Open. 2025 Apr 10;15(4):e094753. doi: 10.1136/bmjopen-2024-094753 (PMC11987160; doi:10.1136/bmjopen-2024-094753)
Supplement: online supplemental file 3 [file bmjopen-15-4-s003.docx]

**Supplementary File 3: Topic Guide – Service providers**

*Suggested probes are outlined below, to be utilised where appropriate, and only if needed to promote discussion*

**Roles and Responsibilities**

- Can you tell me about your current role? How long have you been in this position?
- Can you tell me about your role(s)/experience(s) in relation to miscarriage/recurrent miscarriage?
- Have you received any specific training to support the provision of care to those who experience recurrent miscarriage?
- If you do not have a direct role in providing care for recurrent miscarriage, do you see a potential role for you/your profession and what might this look like?

**Defining Recurrent Miscarriage**

- In your experience, how is recurrent miscarriage defined?
- In your opinion, what is an appropriate definition of recurrent miscarriage? Why?
- What is the impact of the definition on the care provided to those who experience recurrent miscarriage?
- What is the impact of this on the women and men who experience recurrent miscarriage?

**Structure of Care**

- What informs current practice in relation to recurrent miscarriage in your area? (evidence, local/national guidelines, professionals)
- Who is responsible for the decision-making/organisation/co-ordination of recurrent miscarriage care?
- How is funding/resources allocated and what is the process (decision making/stakeholders/timelines)?

**Management of Recurrent Miscarriage (diagnosis, investigation, treatment, management)**

- Can you tell me about the current management of recurrent miscarriage?
- How is recurrent miscarriage diagnosed? Where, when and by whom?
- What happens following a diagnosis of recurrent miscarriage (procedure/pathway/timelines)? Does this differ for different people? (public vs private/age/number of losses/cultural differences/socio-economic status)
- Can you tell me about information/advice provided, if any? Is the information provided appropriate/sufficient?
- Can you tell me about the investigations offered, if any? Who is involved in the decision-making about investigations? Where do these investigations take place? What is the procedure for investigation and follow-up?
- What happens if investigations are inconclusive?
- Can you tell me about the treatment options available? Who is involved in the decision-making about treatment? Where is treatment provided and who is responsible for overseeing treatment?
- In your experience, what are the outcomes for couples following investigation and treatment? (sustained pregnancy/further loss)
- What happens in the case of a further pregnancy loss?

**Impact and Support**

- In your opinion, what is the impact of recurrent miscarriage on those affected? (Women, men, children, extended family)?
- In your opinion, how do couples experience pregnancy after loss?
- In your opinion, how does current care (the diagnosis, investigation and treatment process) impact those who experience recurrent miscarriage? (the process/the system)
- In your opinion, what are the support needs of women and men experiencing recurrent miscarriage? Do these differ by case/demographic/care pathway? In your opinion, are these needs being met? If not, what is needed to improve this?
- How does the current system impact you and/or other health professionals? What is it like to provide care in this context?

**Knowledge and Understanding**

- In your opinion, do couples have adequate understanding of recurrent miscarriage (the condition, the impact, investigations, treatments, outcomes)? if not, why is this the case and what could be done to improve this?
- What information is provided in the maternity unit regarding diagnosis, investigation, treatment, outcomes, future pregnancies, available supports?
- In your experience, is this information sufficient?
- Do women and men look for information and advice elsewhere about recurrent miscarriage? Where do they look? Does this differ by demographic? What is the impact?

**Recommendations**

- What are the factors that make it easier to care for those who experience recurrent miscarriage? What works well? Can you provide examples of good practice?
- Are there any barriers to providing care for those who experience recurrent miscarriage?
- Is there anything you would do differently?
- What would ideal care for recurrent miscarriage look like in your opinion?
- What is needed to achieve this?

**Is there anything that we haven’t touched on that you feel is important to mention? / Do you have anything else to add?**
