# Supplementary material for: Views of knowledge users on recurrent miscarriage services and supports in the Republic of Ireland: a qualitative interview study
Source: BMJ Open. 2025 Apr 10;15(4):e094753. doi: 10.1136/bmjopen-2024-094753 (PMC11987160; doi:10.1136/bmjopen-2024-094753)
Supplement: online supplemental file 4 [file bmjopen-15-4-s004.docx]

**Supplementary File 4: Reflexive thematic analysis approach following Braun and Clarke**[1]

| **Phase** | **Description** |
| --- | --- |
| **1: Familiarisation with the dataset** | We (RD and MH) immersed ourselves in the data by reading and re-reading transcripts and listening back to recordings. This was undertaken as part of checking transcripts against audio-recordings but also separately as we re-familiarised ourselves with the data over the course of the project – returning to the ‘raw’ data at various points during the analysis. We noted observations, insights, and questions about the data (each interview transcript and/or the dataset as a whole) within our researcher diaries and also as annotations and memos in NVivo. We adopted a team approach to the analysis. While coding was led by RD/MH, other members of the team (SM, ROSL, KMS) also familiarised themselves with the data. Each were assigned a number of transcripts, and these were discussed during a series of meetings with RD and MH. Throughout all phases of the analysis, discussions amongst team members aimed to generate deeper and richer insights into the meaning of the data, not to achieve consensus of meaning. |
| **2: Coding** | RD coded the service provider data initially - working through these interviews, identifying data that appeared relevant to the research question, applying analytically meaningful codes. Codes captured single meanings or concepts and included both semantic (explicit or surface meaning) and latent (implicit or conceptual) codes. Throughout the coding process, codes were generated, reviewed and sometimes revised/refined/merged as coding progressed across the dataset. This involved a process of back and forth between the raw data (transcripts) and the codes (‘nodes’ in NVivo). RD then coded the data from interviews with men/partners, and MH subsequently coded the data from interviews with women as RD was on extended leave. Both were cognisant that the service provider interviews were coded in NVivo first and were reflexive around this as they coded the ‘parent’ interview data. |
| **3: Generating initial themes** | During this phase, RD and MH started to identify shared patterns of meaning (initial or candidate themes) across the dataset. They began to bring clusters of codes together that shared a core idea relevant to the research question. This process involved much discussion between RD and MH and the broader team. While NVivo was used to manage data, the process of generating initial themes also involved drawing concept maps – by hand, in Miro (a visual online workspace) and also writing initial summaries of candidate themes. MH then collated all relevant coded data under each candidate theme. |
| **4: Developing and reviewing themes** | MH then reviewed the candidate themes against the raw data. This involved checking that the themes made sense in relation to the coded extracts and then the full dataset (full transcripts). It also involved assessing the relationship between the themes, the existing literature, lived and clinical knowledge, and the broader context of our research. We did not radically revise our themes at this stage; instead, we made some tweaks. |
| **5: Refining, defining and naming themes** | In this phase we refined our analysis through writing summaries of each theme, naming themes, and reviewing and editing these. We reviewed each theme, ensuring that it stood apart from the other themes and was built around a core concept. We constructed themes that we felt got at the core of the issues contained within the underpinning narratives. |
| **6: Writing up** | During Phase 3 we began to formally write up our analysis, generating initial descriptions of our initial themes. We had been writing informally up to that point – through familiarisation notes following interviews; notes taken during debriefings with the other interviewer (RD and MH conducted interviews and spoke with each other after each); notes taken of meetings with (i) the wider qualitative research team (RD, MH, SM, ROSL, KMS), (ii) the Research Advisory Group, (iii) the Project Management Team (RD, MH, KOD) and (iv) people with lived experience who were also members of the Research Advisory Group and co-authors (JUD, CL). In Phase 6, we drafted a full version of our analysis – bringing our analytic narrative coherently together, supported by illustrative quotes which we have made available in tables due to manuscript word limit constraints. |

**Reference**

1 Braun V, Clarke V. *Thematic Analysis: A Practical Guide*. London: SAGE Publications Ltd 2021.
