# Supplementary material for: Views of knowledge users on recurrent miscarriage services and supports in the Republic of Ireland: a qualitative interview study
Source: BMJ Open. 2025 Apr 10;15(4):e094753. doi: 10.1136/bmjopen-2024-094753 (PMC11987160; doi:10.1136/bmjopen-2024-094753)
Supplement: online supplemental file 5 [file bmjopen-15-4-s005.docx]

**Supplementary File 5: Illustrative quotes for Theme 1 | Dedicated staff**

| **Sub-theme 1.1 \| Dedicated staff have specialist and experiential knowledge** |
| --- |
| “I’d spent a week so upset and going what will I do [go public or private in subsequent pregnancy] and would I be better just going publicly so I’d be going through the hospital and I know *Bereavement Midwife* is there and I know the sonographer is really good and I know the other midwives are there and they’re really good. You know I felt really safe when I was in with them last year. Really safe. And I’d collapsed during the night in the toilet and there was suddenly five people standing around me. I was going oh my God I’m dying. But I still felt really safe.” (PW3) |
| “You went in, and you saw either herself [the consultant] or her registrar, but it was the same people you were seeing. It was only her or that other doctor. There was the same nurse that you saw… They know your name when you walk into the room, and they read your notes before you walk into the room. So at least they know a little bit of your story before you go in. And you don’t have to repeat yourself 24/7. So I went in there. I found her care was, she was much kinder and much more understanding and empathetic probably.” (PW11) |
| “You have that empathy from the person we’ll say. They know your story. They know what this means so they know how to handle the situation I suppose.” (PM1) |
| “I think if we didn’t have a dedicated clinic, I think a lot of these women would be falling by the wayside a little bit I suppose. I think any maternity hospital, if there was a certain number of patients, kind of needs to have this service, but it needs to be a service delivered by somebody with persistence but also with expertise. And the expertise can be garnered from working in the clinic over time…” (OBGYN3) |
| “Unless they’re going privately or unless they’re under some dedicated consultant in recurrent miscarriage who can offer that [reassurance scan], and most of the clinics don’t, you know, they end up going in through an emergency room to see a random doctor on duty, who may or may not be able to scan. They can often end up with over intervention because they’re coming so early when a pregnancy is too small to determine what’s going on and they end up with two or three weeks of follow-up while its being determined is it viable or not. And they end up coming through early pregnancy units and long waits and bad associations from the previous times they were there. So I think it is hard for these women. I think they are forgotten a lot of the time because they are not huge numbers, they’re not, compared to people who have had a fetal death, or you know who’ve had preeclampsia, early gestation or whatever, it’s not as glamorous. It’s not as high-profile research-wise or academic-wise, or even in industry, there’s nothing. There’s no drug they can market that’s gonna improve all of this.” (OBGYN4) |
| “… the benefit of having a clinic is that if you have people that are working in an area obviously for a long length of time, they’re going to have experience and expertise. And that is important. And I know all clinicians should have a knowledge of miscarriage and all of that. But I’m just saying if every week you have a clinic even for myself you gather so much information over the number of years I’ve been in the service you know. You have an awareness of what might be needed for the women. You have an awareness of where to direct them… So you have a confidence in what you’re saying if you’re doing it. And it’s like anything, you build up expertise.” (BM2) |
| “… often times we have a relationship built with these women already. We might know them you know. So they have the freedom to contact us. Some people then won’t. And some people we know for a long time they might have had ten miscarriages and no baby.” (BM8) |
| “It very much depends from hospital to hospital. It depends on the culture around bereavement care I suppose and the level of importance that it has within that particular organisation. Culture has a lot to do with it I suppose, organisational culture.” (AGM1) |
| “I think if we had a dedicated pregnancy loss service in each hospital where there was just a dedicated person who was you know ensuring that people were followed up appropriately and investigated appropriately and that they were then given support in the next pregnancy and just that there was I think better ownership over everything as opposed to the somewhat chaotic services that we can have.” (SPR2) |
| “And there’s nobody [in the gynae clinic where women with RM are seen] who has really a special interest which isn’t a great thing. And to be honest, they wouldn’t be the most empathetic of them, yeah. And the thing is the clinics are so overrun that really they could be seen by a reg or an SHO [Senior House Officer]. You know, there should be a dedicated clinic I think for women with this problem, but there isn’t unfortunately here.” (GP4) |
| **Sub-theme 1.2 \| Staff need (ongoing) support and training** |
| “I was brought back in after six weeks… *Consultant* wanted me to come to the public ward. I don’t know. I think it was a misunderstanding between us. But she was like, ‘…and *Bereavement Midwife* will be there and you’ll have *Bereavement Midwife*’. But when we went there, *Bereavement Midwife* wasn’t there, and we were just in the original outpatients clinic, and a young intern doctor had started the week before. And all my records were electronic so there was just a sheet placed on top of a folder with my name on it. So he just took the sheet and the folder. And then he opened the folder and went so we’re going to talk about your polycystic ovaries. And I go, ‘no, I don’t have polycystic ovaries’. And I got really upset because I had it all built up. I wanted to know why my baby died… And they were supposed to send the remains for some kind of genetic testing and they decided not to, and I wanted to know why that had happened. And he just got more and more confused, and I got more and more upset. And then he went out and got his SHO, who actually had been with me on the night, and she’d been really good. And they eventually ended up getting… there was a locum covering the clinic that day and he came in. *Husband* said afterwards you brought the whole clinic to a stop you got so upset.” (PW3) |
| “Like some of them [midwife sonographers] give so much time and it’s not just oh sorry this hasn’t worked out or whatever. Some have really given that time. But again, they obviously need supervision and support to keep going in that environment.” (PW7) |
| “But I have to say another thing then too about the midwives, is that that can’t be easy on them either, you know.” (PW9) |
| “We would never allow somebody who isn’t versed or hasn’t done a clinic before, go in you know to a couple and just see how they get on. You know we’d never do that. So it has to be people… it would never be somebody who is in their first year of training whose never done a thing. So we’re pretty strict that it has to be somebody with experience and knowledge. Because, as you know, these people have been through a lot. It can be hugely morbid for them and psychologically, and they can be very angry, and they can be very enquiring, and extremely cross when they find that actually there’s nothing in their test results you know. So I always have to tell the junior doctors who are with me, you know, you have to train them up and just give them a little bit of advice if it’s their first time being in the clinic.” (OBGYN2) |
| “You know so I suppose people have their own areas of interest and sometimes they just inherit the miscarriage clinic when they take up their post and it wasn’t really what they loved doing… For me I was just lucky and I just kind of… I love it now that I have the bereavement liaison midwife team. I love it now because they’re just brilliant people to work with you know. So I suppose at the route of, coming back to your question, you know what makes it successful or unsuccessful in an institution, I think the fact that it gets proper billing and it’s a recognised service and it’s a department you know within the hospital. Yeah.” (OBGYN2) |
| “…I think, you know, what would actually be very useful is if the bereavement midwife did more in teaching training I think at the beginning of your rotations in the hospital. You know, you just get the one induction day where everything gets thrown at you and generally all of pregnancy loss you know gets a tiny timeframe. So, you know, for the bereavement midwives to cover anything is impossible in that tiny timeframe. You would really need a separate teaching session. And I mean that kind of does happen sometimes, but it tends to be a bit haphazard you know who organises it and when and who turns up for the teaching and what is covered.” (SPR1) |
| “I think she [CMS in Bereavement and Loss]’s so committed and dedicated to what she’s doing. And she certainly has made a significant impact in terms of her role both amongst the staff in terms of confidence and for women. And also in terms of education induction. We’ve actually increased the amount of sessions we’ve had just to have more of an impact in terms of confidence amongst our NCHDs [Non Consultant Hospital Doctors] and that’s happened this year. So we’re just going to see how that works out. But she’s certainly been a huge addition to the services here.” (DOM2) |
| “People forget the impact they have on lives and, you know, what a word can mean, or being dismissed or being hurt. Because when you’re vulnerable, you know your radar is very different…” (BM2) |
| “…we put on, we do kind of in-house training and kind of on-the-spot training, but that’s not enough. There’s only one of me, so I can’t get everywhere. Do you know what I mean? So we did, the first year we did a one day training program which actually was really good and was really a big success actually. And it touched on a lot of different you know the practicalities and then the emotional, and always in those cases the most effective is actually having members of the public and their experience. It’s always the most effective.” (BM3) |
| “I’m in the group of, national group for clinical midwifery specialists in bereavement, and we meet up as much as we can throughout the year or whatever and we communicate through email, phone calls, all that kind of stuff. And we pick up a lot of support, advice, information from each other. And that helps a lot.” (BM5) |
| “No matter how long in the game you are, or no matter how well prepared you are, it’s difficult, you know.” (BM7) |
| “And I think for staff who experience and witness losses like that all the time it can nearly become a normal or there’s a kind of a normalising of it because of the coping…… It’s important to mind the staff because they’re dealing with it every day.” (BM8) |
| “I would love to have cooperation with other clinics around the country. I would love to see meetings and you know exchange of information and ideas between, you know, the clinics that have an interest in this, so *Hospital 4* and then the other maternity hospitals in *County 6,* and anywhere that runs a good service. I would love to have a network of interested people to collaborate and to maybe do meetings and discuss cases. I would love that. Because I do feel a little bit it’s up to me to get this right. There’s nobody else in *Hospital 2* who’s interested in this or who can disagree or agree with what I’m doing, you know what I mean.” (OBGYN2) |
| “… it can be an unrewarding subject to look into because in many cases you cannot find anything to help…… In fact, in more than 90% you probably don’t find anything that you can treat or change. So that’s what I mean by unrewarding. It’s not that you can’t extend sympathy and be supportive. But it means that in actual factual terms can you stop the problem from happening, very often there’s nothing you can do…… I mean most doctors and obstetricians are relatively sort of practical people. If you come with a problem, it’s good to send the person away with the problem sorted. And that is not necessarily the case with recurrent miscarriage.” (AGM4) |
| …recurrent miscarriage is not neat. It’s not tidy. And it’s not nice. Pregnancy is not supposed to end up in loss. Pregnancy loss is not nice because it is such an abnormal phenomenon. And that goes for midwives who would have years of experience, they don’t like it. I don’t like it… But it happens and somebody has to support these women.” (BM4) |

| **Sub-theme 1.3 \| Opportunity to better connect with primary care providers** |
| --- |
| “… the GP doesn’t seem to be involved in relation to the care about miscarriage because they see you after you have a baby. There’s no kind of... Nobody tells you that you need to go to your GP or anything, because you don’t I suppose really. But they would have had limited... Now I do have a good relationship with my GP, but like she would have had limited participation in the whole area of miscarriage and involved in my care for that.” (PW1) |
| “I know I would have gone to A and E [Accident and Emergency] after taking the medication management a couple of days later because I was feeling really weird. And there was no letter sent from anybody to my GP. But I think all the other stuff had been fed back to my GP. She had been really supportive actually. I remember when *Baby 1* died she rang to see was I alright and suggested I go for counselling you know.” (PW3) |
| “There’s no actual kind of formal structure really as I’d see it… Now I either do the blood tests or else the people will get the appointment, you know they’ll be told. But what I’m finding is they’ll get the list of bloods and then when we have the blood tests and they get them back and they’ll give an appointment. But sure that’s very kind of shall we say too unstructured really. So where am I sending the blood tests, who’s going to pick up on them you know. I find that kind of challenging myself……… Sure I don’t know where it’s going. And if I send in the results who’s going to pick up on them.” (GP1) |
| “… we know our patients for a long time generally and we kind of go through the whole antenatal process with them. And that rapport is really helpful when we try to support them when things go wrong. So communication both ways is really helpful………… *Hospital 10* is our local hospital so it’s just down the road. So we’ve got a good relationship with them. They’re great. Like for the most part people have a very positive experience there. But sometimes just things fall through the cracks you know, everyone’s busy. But having that standardised communication makes… would make a big difference… So if we get a letter like that [from the hospital about a patient’s miscarriage], obviously we read all of our post and then we’re able to pick up the phone and just check in with the woman and say you know how are you, I see that you’ve had a miscarriage and just kind of close the gap. Because it’s an awful thing for her to otherwise have to contact us the next time and for us to not know. You know that can be really awful……they’re very appreciative. And the other benefit of that is that we can cancel… so women will have been enrolled in the mother and infant scheme and if we don’t know that the pregnancy is no longer continuing they might get contacted by the PCRS [Primary Care Reimbursement Service] you know the government agency with a letter in appropriately. And so we can kind of put a stop to that as well. So, yeah. The communication is super important.” (GP3) |
| “I would refer in [to the recurrent miscarriage clinic] and do the bloods, and work them up and that. Yeah. Because you will have you know a lot of people who just have the early miscarriage and maybe never had attended the hospital or the early pregnancy unit, you know.” (GP4) |
| “… maybe if we could get someone to come and speak to us even just to talk to us about what tests are done, recurrent miscarriage is actually investigated, is it after two, after three... Because, you know, okay, you might not come across somebody for a long time. It could be a couple of months. But even having that information in the back of your head you can always pull from it, you know. So I do think even us knowing what actually happens in the hospital for them, and what they’re experiencing in the hospitals, would be good for us you know…………I do think the lack of education would be the biggest one really, you know. I suppose your own lack of knowledge is obvious then.” (PN1) |

Note: AGM: National Administration, Governance & Management, BM: Bereavement Midwife [Clinical Midwife/Nurse Specialist in Bereavement and Loss], DOM: Director of Midwifery, GP: General Practitioner, OBGYN: Consultant Obstetrician/Gynaecologist (Hospital-based), PM: Man who has experienced RM, PN: Practice Nurse, PW: Woman who has experienced RM, SPR: Specialist Registrar.
