# Supplementary material for: Views of knowledge users on recurrent miscarriage services and supports in the Republic of Ireland: a qualitative interview study
Source: BMJ Open. 2025 Apr 10;15(4):e094753. doi: 10.1136/bmjopen-2024-094753 (PMC11987160; doi:10.1136/bmjopen-2024-094753)
Supplement: online supplemental file 6 [file bmjopen-15-4-s006.docx]

**Supplementary File 6: Illustrative quotes for Theme 2 | Dedicated space and time**

| **Sub-theme 2.1 \| Lack of dedicated or appropriate spaces** |
| --- |
| “I was sitting on a hard chair on a busy corridor of a maternity ward outside the kitchen door having been told that I lost my baby. I was there alone. Nobody to offer me a word of comfort. Nobody to… I just felt like, oh my God this is so wrong. Which it is. I mean it’s so bad…… I must have been waiting close to three hours. And eventually I said to the midwife I said I’m leaving I said I can’t wait anymore you know I said this is just too upsetting. I said its actually inhumane to leave a woman like this.” (PW1) |
| “But I found then when I went in and when I was having my section here it brought every loss back. Because I walked into the theatre like I walked in every time. Sure I completely lost it. They actually stopped everything and scanned me because I’d say they were worried because I couldn’t talk, I got so upset.” (PW2) |
| “[my wife] was brought up to where the rest of the women were having their babies. And she was put in a room beside them to lose hers. Which is, I think is completely wrong.” (PM3) |
| “We have a designated physical space. It’s not ideal, but we protect them as best we can…… away from pregnant bumps because, as I say, the antenatal clinic is over at that stage. They might see someone waiting for perinatal health there, but they don’t know why they’re waiting there.” (BM8) |
| “Like infrastructurally we’re in a really old building. It’s really terrible. And where we used to see people, it just was awful, very grim. So they have, you know, little by little, gotten a bunch of money off you know different pots, and they have redecorated the counselling room now, you know, so it’s beautiful. It’s small but it’s beautifully painted and there’s lovely flowers painted on to the wall, and there is dim lighting, and there is, you know, it’s just those sort of touches.” (OBGYN2) |
| “I think the environment in which news is relayed is very important irrespective of the delivery and style and content. You know I think the environment is very important. I’m not sure that we have that right either.” (DOM2) |
| “These are the problems that are with the maternity services you know kind of in a more general way. You know big waiting rooms with lots and lots of people and everybody kind of feels very vulnerable and privacy really is the problem. I think it’s all a little bit chaotic and haphazard is the problem. And maybe it’s that, so miscarriage is common obviously and maybe in the maternity services people can get a little bit complacent about it because it’s so common to them but of course this is a whole new language and experience for women who are experiencing it for the first, second, third time...” (GP3) |
| **Sub-theme 2.2 \| The importance of giving time, yet time is constrained** |
| “So I remember after the third one… and of course I’d be in contact… I’d ring the bereavement midwives and you might or you mightn’t get them. Now I say that very respectfully because they were brilliant ladies, but they just cannot keep up. I’ve been with them when their phones are ringing. I’ve been in the wards when the midwives are trying to get them. I’ve seen how tired they are. And I’ve seen them trying to, they’d even referred me to counselling services in the hospital because they just can’t be there for you.” (PW2) |
| “I got the impression that *Consultant* was delighted to get rid of me. I don’t know. He just seemed very busy, and all his women were pregnant and he just… I just got the impression that he was glad it wasn’t a problem he had to investigate, you know.” (PW4) |
| “But it would be nice if you did meet *bereavement midwives* in that first time [after having a miscarriage]. Then you’d be more likely to ring them back instead of being at home and feeling lonely and not having that contact. That you could contact them then to say, ‘oh look what do I do next’……Yeah it would be easier to do that phone call. Otherwise, you’re ringing up like it seems like a helpline number.” (PW7) |
| “… it’s definitely been a really trying experience [recurrent miscarriage]. But for me, the people that took the time and, you know, had that certain approach will always be the real people that helped me along the way.” (PW12) |
| “It was just kind of like, ‘right, well this is what I have to give you and we’ve done our bit and I have to go’. And I do know they are busy, but it was just a bit abrupt when they knew already that the baby was gone. I do feel like they could have had you know five minutes kind of maybe to say is there anything… because there was never really that time either.” (PW14) |
| “Yeah, it can be frustrating at times when, you know, you’re trying to chase up a consultant to get results looked at and it could be a week before she looks at them or it could be two weeks so you’re ringing her again to look at them or whatever or will I bring her back to the clinic or what. You know that’s where there’s a lot of frustration for me anyway and I know the other two nurses when you’re trying to get results…” (BM1) |
| “…And I suppose as a support midwife I find it really difficult, because I know there are those people who need support and aren’t getting it………… I get exhausted and I kind of wonder sometimes about, I have difficulty in valuing my role I would say. And that’s a really honest thing to say I think because in an area which is so busy the role necessitates slowing down.” (BM3) |
| “But when I was coordinating that [recurrent miscarriage] clinic I used to find it really frustrating. I really was very dissatisfied because I never had time. They met me so they were able to put a face to me and I would give them my card, but I didn’t have time to actually say how are you, like really how are you. So there was no time for a support clinic as well so that was, I found that hugely frustrating.” (BM7) |
| “I would say definitely four to five months before they’ve the bloods done. And then, yeah, it could be six months before they get their appointment, which is a long time. A long time when they’re waiting, and every cycle at that stage is often very important. But the reason I suppose we only have a clinic every fortnight and we only have maybe four to six slots... Because they do take time. When you’re taking a history, they do take a bit of time. We’ve an old hospital. Space isn’t huge. Consultant availability. So, we hold a clinic in the afternoon in the antenatal clinic downstairs. It’s quieter that time. We’re very conscious of bringing women back to that clinic you know when they’re bereaved and it’s hard for them to come back in the first place. So we keep the numbers small. We don’t like them waiting long at all, so we’ve spaced out the appointment times over the afternoon. Yeah. So we only run it every fortnight you know. So that’s I suppose why the reason the waiting list as long as well. And also, we do receive a lot of referrals.” (BM8) |
| “I do prefer the *Hospital 4* approach to it. From my experience there we, you know, we had say, in a morning session, it used to run on a morning session, and we would have maybe had four maybe five patients at most. And it meant that you have plenty of time to dedicate to these patients, because I think they do need time. In *Hospital 1* there were more, maybe we’ll say seven/eight patients booked in that session, and I did feel sometimes it was a little bit rushed as well so I didn’t particularly like that, for myself or for the patients.” (OBGYN1) |
| “…people [with recurrent miscarriage] need a lot of time. And kind of similar to fertility patients when you don’t find anything wrong and they’re so highly invested. I think an awful lot of it is people skills and its dealing with their expectations and its being able to counsel them. You know, a lot of the time there isn’t anything sinister wrong, and you know hopefully things will turn out. The odds are very in favour of successful pregnancy in the future. And it’s just giving them that reassurance I suppose and support, whether it’s, not just a medical intervention but you know physical support as well, and having that early link in I think, and continuity then in their next pregnancy.” (OBGYN4) |
| “Well I think it’s not an emergency in the majority of cases unless you have really a lot of bleeding and then it becomes an emergency and then its supported. But I think I suppose we’re so busy and caught up with all the consultants and all the difficulties and challenges that we have on our wards and you prioritise what’s your high risk and you deal with that. And it’s just that you feel that the women can just get on with this and you know they get over it and they get on and hopefully they’ll have a normal pregnancy the next time. That’s kind of the attitude I think. And I don’t think they realise the impact really that it can have on them. I’m not chauvinistic or anything but there is that type of attitude you know you just need to get on and try again.” (AGM2) |
| **Sub-theme 2.3 \| The need for non-judgemental, empathic, accessible and timely communication, information and support** |
| “They seem to be so flippant about it [recurrent miscarriage], you know. I don’t know, is it just because they’re dealing with it every day as a normal part of their day, a normal part of their job. But like it almost feels like sometimes they seem to forget that there’s a person behind all of this.” (PW1) |
| “The emergency room is one thing. Like when you ring, and you say you’re bleeding, I know they might get a lot of people, but I think telling you to stay at home is really cruel on you to wait. I just do. I do. Now I know when you go in and see, they discourage you, and I don’t think that’s a nice practice. When you go in you will be seen, but you’re discouraged from going in. I don’t know if anyone has ever said that to you before…… I don’t think you should ever be discouraged from going into the emergency room. I think they do it too easily.” (PW2) |
| “I think maybe after you’ve had one miscarriage, they should tell you exactly what to do when you get pregnant again. And what they can offer you when you get pregnant again, and what to say if you think something has gone wrong during that pregnancy. It’s kind of hard to know what to do after having a loss when you get pregnant again because it’s really weird. We’ve been minded a lot which has been really good. But again, we kind of looked for it and asked while last year we didn’t ask. We didn’t ask for any help, so we didn’t get anything because we didn’t ask, you know. But we weren’t steered in that direction by the GP either. Maybe we were and I wasn’t really paying attention to her.” (PW3) |
| “The two midwives I met, the one nurse in the emergency room and the midwife I met in the D and C I just really don’t think they should be giving out advice…… It was especially inappropriate in the emergency room when I had just found out that there was no baby, you know. I didn’t want to know anything about IVF or Aspirin. I didn’t need to know. And then I went into the D and C, every person I met, like the porter bringing me down, and the anaesthetist, they were all great. They were all, the first thing that they’d say was ‘we’re very sorry for this’ or ‘we’re sorry for your loss’, or ‘we’re sorry you’re in here’. And they really looked sincere, and that’s all you need to hear like. It was perfect. There was no need for anymore.” (PW4) |
| “I think in an ideal world *Consultant* should have had me back and should have gone through the results of my other tests with me which he never did and said what was wrong or what was right or what was potential treatment for the other things that might have been wrong with me. I think like definitely after you have an appointment with the early pregnancy unit, or have a D and C, you should be offered a follow-up appointment at a time maybe a month or six weeks afterwards, and you can ask any questions you have about it. I mean if you go to the early pregnancy unit after three miscarriages and they do tests and you’re fine, what do they do then? (PW4) |
| “I asked my GP for a referral, but she was reluctant to refer me, because she just basically said, ‘look, you know, it’s your age, they can’t do anything for you’… I was very disappointed obviously [to get that response from the GP], and you know it’s horrible to have it blamed on yourself.” (PW5) |
| “… a lot of the individuals are very nice, you know, a lot of the midwives and the doctors have been very nice. But I think as a whole, the whole service needs to be a bit better I guess in terms of being a bit more, how do I say it, kind of just united in terms of communication, and what kind of service they’re offering…… There’s so many different lines, you know, I don’t know what the truth is. And I just feel the service is disjointed, the whole thing.” (PW5) |
| “I had the fetus in my bag, like literally in a box, but no-one asked me, and I didn’t want to say ‘here it is’, you know.” (PW10) |
| “And I have to say the only information I’ve ever really gotten is off the forums on roller-coaster or on fertility friends which is horrendous. Because I’m not even on Facebook. I’m not one of those people. Like I’m not a social media dependent person. But yet this is where I’m… And that’s where I’m getting my information from…… Because I had to go there to look at was there any other treatment options, could I do anything.” (PW11) |
| “But I suppose my experience throughout the whole thing has been that it can be a very, very lonely place to be in terms of going to these clinics, going to meet people. You’re really putting yourself out there. You’re very vulnerable. And for me the hospital, actually in *County 1,* were the people that picked up on that the most. You know they were always very, very good and very aware of the significance of it.” (PW12) |
| You know so it was very traumatic physically and emotionally [the first miscarriage] but like I think where the services could improve would be definitely, don’t play things down. Empower women to have knowledge of their own bodies, and don’t hide from them the stuff that could go wrong with their bodies. You know. Like it’s just going to be like a period. Like it’s not. That’s not correct. Well, it wasn’t for me.” (PW13) |
| “We got given a booklet by one of them on the first miscarriage, and she was just like ‘this is a booklet for your information and there’s different numbers if you wanna call’. And I did call one of the numbers, but I never got any answer. *Laughs* So then I kind of thought, well I’m gonna upset myself more trying and not getting an answer than just not trying, so I just didn’t bother again.” (PW14) |
| “And in fairness to the *County 1* service they have their website that’s really good. You know that’s fact-based information. Because obviously you can go down rabbit holes. You basically have the extremes you know. You have the people – is it Rosanna Davidson who had fourteen miscarriages and now she has kids? You’ll have loads of stories online of people, oh I did this and then I was able to have babies, and you can get lost in those stories. But it was good for me to have that website. I think it’s really good…… So I think I had looked at it before we ever talked to *Bereavement Midwife*. So yeah, I think that was good to just have the one stop shop that said look this is how likely it is. Okay, it’s not what you want to hear but when you’ve had miscarriages you are more likely to have them. It was just good because you have, yeah, the fact-based information. You’re not giving yourself false hope by looking at a load of random stories I suppose. And you’re not getting too dragged into the doldrums by reading other stories and bad stories suppose.” (PM4) |
| “I do feel – and *Wife* might disagree – but I feel that *Wife* could maybe have benefitted a little from it [counselling] to seek it. I suggested it to her and she kind of said maybe. I do feel like it was great meeting *Bereavement Midwife* and *Consultant,* but I feel that that’s maybe one addition that could be made to the service is… I haven’t actually heard of the Miscarriage Association up until you mentioned it just there. I think it could be good yeah to just have… You know, when it’s like that, when it’s just me recommending to *Wife* it’s one thing, but if you had someone like *Consultant* or even any of the doctors in the maternity hospital that said, look, I don’t know is there specialist counsellors, or even just look here’s a list of five counsellors in the local area that specialise in grief or that specialise in miscarriage, would be amazing but I kind of doubt that’s there.” (PM4) |
| “I think the timeframe I suppose people when they have the tests done, they want the answers there and then, and it’s just not the way it is. So people find that difficult. So I suppose again it’s all about setting expectation and saying ‘look, this could take eight weeks or this could take six weeks’. And I think if they leave the hospital knowing that that can make it easier.” (VS1) |
| “I suppose there’s always room for improvement in every service, but I just feel we’re losing out on our referrals. If we could get a proper referral system you know it could be quicker, things would be more efficient.” (BM1) |
| “But like that if the results aren’t back on the week they were told they’d be back they get upset, they get frustrated, they get cross. But I suppose I can understand the pressure they’re putting themselves under because, you know, they’re anxious to get pregnant again. Another month is gone, and another month has gone, another month has gone, you know, and it builds up a lot of anxiety in them. (BM1) |
| “And you also have to be mindful [when referring someone to an information source] and I would always say that to them even if I was still referring them to that site I will always say look you need to mind it. There will be things mentioned in that site that are not applicable to you.” (BM2) |
| “When they’re in the hospital [after having a miscarriage] they’re in an acute state of trauma. So they don’t often hear, especially if they’re recurrent miscarriage. They are in really kind of in that suspended state a lot of them. So their ability to reach out is… And then when they go home they have forgotten the numbers or the contacts you know…… So I think that’s perhaps some of the reasons why you know that they kind of get missed [by the clinic].” (BM3) |
| “And there’s age related discussions as well you know about the risk of, you know, genetic problems increasing as you get older and all that kind of stuff. But it might not always be that well-received… You just have to support them in whatever decisions they make, you know, and give them the best advice that you can in order to make that plan for the future. And, you know, they might have a clinic appointment with *Consultant* and myself, and I might get a call a week later saying well, you know, *Consultant* advised me to lose weight, or gave me information about the increased risk for genetic disorders as you get older, and things like that, or they didn’t understand a table, or something like that, and they want explanations, or further advice, or, you know, can they get in touch with *Consultant*. So, you know, you communicate with the consultant and she may actually give them a follow-up call then just to clarify those issues.” (BM5) |
| “I think it’s variable [information that’s available to parents] would be my answer really. Because you know Google could be your best friend, but it could also be your worst friend I think really. So I suppose when you mention that there probably is a need to kind of have a dedicated source for patients to look at rather than the random Google basically because that’s always where they come up with all sorts of different ideas.” (OBGYN1) |
| “I do offer them booking into, so basically what we say is, if they opt for aspirin or progesterone for example, I give them the prescription there and then so they’re ready to start... When they do conceive again, they have *Bereavement Midwife*’s phone number. They have the early pregnancy unit’s phone number as well. So we organise an early pregnancy scan for them, and I offer them to book into my antenatal clinic basically. So there’s more of a follow-on I suppose really. I think that’s important for the patients as well. You know you’ve developed a rapport with them, and they have that trust and confidence in the team basically, and to follow that on as well in early pregnancy I think is really important cos they… That support even has been shown as well to sort of improve their experience in the next pregnancy so.” (OBGYN1) |
| “Once you take the time to explain that to people, they understand, you know. They are always thankful of having the explanation. We send them… We have a very nice booklet on the kind of causes of recurrent pregnancy loss, the investigations that are done, so its lovely to have that to just be able to send people. They can sit in the comfort of their own home and have a cup of tea and read it and then understand it.” (OBGYN2) |
| “The referral yeah, most people are very happy with the referral [to the recurrent miscarriage clinic], if they’re eligible for it. There’s been very rare cases where people don’t want it. Occasionally, there’s a lot of people who are non-English speakers as their first language where I’m working, so probably at least two out of the fifteen a day we’d have to use the translator services. And I think that’s harder when you’re trying to do that. So you’re via speaker phone and you’re trying to socially distance and tell this and you’re trying to explain about you know miscarriage and options. And then kind of I think the whole thing about recurrent miscarriage comes at the end of that, and it’s a bit exhausting for all involved by the time you get down to that. So I think probably it’s not done as well in those patients. Yeah, so I think there’s potential that some of those are missing out really.” (OBGYN4) |
| “I think you have to be careful about how you approach the conversation. I think you have to kind of maybe say sensitively that we know that obesity can be a factor in miscarriage you know, were you aware of your weight, do you know what your BMI is, do you know what sort of weight you’d need to lose to get that BMI, do you want help with that? You know, you need to try… It’s a difficult balance between trying to put some responsibility back on them without making them feel that they have caused them to lose their baby…” (SPR2) |
| “… it’s just kind of a judgment on the day. You know, somebody may come in and they’re very tearful, and the last thing you want to say… So on the one hand you’re saying ‘oh this is nobody’s fault, there’s no reason for this’. You know, you don’t want to start turning around then and saying ‘but actually if you do X Y and Z, it may improve your chances’. And there’s no great evidence towards that anyway you know.” (GP4) |
| **Sub-theme 2.4 \| Recognising and meeting the needs of men/partners** |
| “I suppose, you know, you are there to support *Wife* as well. You know I can’t do anything. I can’t go through the miscarriage. I can’t go in through the D and C. I can’t take the tablets. I can’t have the feelings, even though I’d love to kind of, you know, to take a bit away. But you can’t. So then you’re trying to support them.” (PM1) |
| “*Wife* does I think a monthly support group Zoom. Me myself I just, I don’t. It wouldn’t benefit me I don’t think. I kind of grieve. And I’m not trying to be like a toxic masculinity macho man. Like that’s not me. It’s not my personality. If I thought it would benefit me, or even if I was compelled enough, even if I didn’t think it would benefit me, I would probably give it a go, but I’m more I think my mind frame is more looking to the future. Like I don’t wanna give up, you know. I think my mind frame is already on to the next one, which I know is not what a woman’s mind frame could be, you know.” (PM2) |
| “It did put a strain on our marriage alright. Because I’d come in maybe after a day’s work. I’d be thinking about it all day, like that I wouldn’t talk. Because I… I wouldn’t bring it up. Because there’s days I see *Wife* there, and she’s doing so well, and I don’t wanna bring it up to set her back. I don’t wanna bring it up to bring back, you know, emotions. So that’s one of the reasons I’d sort of hold it, hold it in, and not talk to her about it…” (PM3) |
| “I think if he’d asked I think the bereavement midwife would have met with him. She said herself ‘we’re not great with the dads’. And I’m not sure. So when you’re in hospital, and you’re the emergency, you know, and you’re the medical emergency. I don’t know. The focus seems to be all about me and making sure I’m okay and not dying or anything.” (PW3) |
| “I went to all the appointments on my own. I don’t mind going on my own. I didn’t want him to really go. I probably take it in more than he would. Like it would go over his head. He just came in to get the blood test for *Consultant* and *Consultant* rang me first even though my husband was involved. He rang me with the result of it. He did ring him and tell him the result as well, but I suppose he knew it was me it was affecting. But he had no other interactions. Nobody has asked I suppose, and I didn’t feel the need to have him. I know some women would have their partners at every appointment, but I didn’t want that or need that I suppose.” (PW4) |
| “… sometimes they [men/partners] end up taking a back seat. And some of them can be quite upset and emotionally traumatised by, you know, even just the whole process of miscarriage even if it happens at home, you know. Their partner going through pain and bleeding, and they feel helpless. And, you know, they have mixed emotions. They go through sort of like feeling guilty that they’re putting the person through it, you know. And they grieve differently………… it’s not that they don’t matter but just that their engagement is slightly different ……… Because for women I suppose we’re much better at talking about it. Men don’t often have the same network of support. Their buddy in the pub and there might be someone in the soccer group or whatever they play, or they talk about it, but they tend not to be as good emotionally about expressing it. So they can feel quite isolated that way, you know.” (BM2) |
| “Yes they [men/partners] definitely would come in [to clinic appointment]. Well not definitely, but at least at one visit they would come in to the designated with *Consultant*. Yeah. Absolutely. And I think that’s really important. I mean the male factor for the support, plus for them to know what’s going on. And actually, plus I believe it’s very important for us to get an idea of what’s the sense of them as a couple. And that actually informs my support to them out of that. What I would say about it is, I find as a support midwife, how to support the husband in the loss and the support of their wife in the loss is really difficult. And I think it is really a big area that needs a lot of work to build on and you know maybe we need to get more information about men. I find it’s really difficult for many reasons. I mean biologically they experience, you know, that’s just nature. And their focus really, especially in miscarriage in the beginning, especially if there’s traumatic miscarriage, you know, if they bleed a lot, some are freaked out that they’re losing their partner never mind anything else, so that’s their focus. So, you know, it’s all that really intricate kind of emotional knitted ball and it gets very difficult. And it gets very difficult for their relationship. So like it’s all huge. You know it’s really huge. And so the dads, for the emotional support, I don’t tend to see a lot of them in the miscarriage, you know, the early miscarriages anything under the twelve weeks… I could probably count on one hand the amount of times a man has come in for that support.” (BM3) |

Note: AGM: National Administration, Governance & Management, BM: Bereavement Midwife [Clinical Midwife/Nurse Specialist in Bereavement and Loss], DOM: Director of Midwifery, GP: General Practitioner, OBGYN: Consultant Obstetrician/Gynaecologist (Hospital-based), PM: Man who has experienced RM, PW: Woman who has experienced RM, SPR: Specialist Registrar, VS: Community & voluntary sector organisation.
