# Supplementary material for: Views of knowledge users on recurrent miscarriage services and supports in the Republic of Ireland: a qualitative interview study
Source: BMJ Open. 2025 Apr 10;15(4):e094753. doi: 10.1136/bmjopen-2024-094753 (PMC11987160; doi:10.1136/bmjopen-2024-094753)
Supplement: online supplemental file 7 [file bmjopen-15-4-s007.docx]

**Supplementary File 7: Illustrative quotes for Theme 3 | Dedicated funding and support – prioritise recurrent miscarriage**

| **Sub-theme 3.1 \| Multi-level buy-in, collaboration, leadership and champions needed to affect change in recurrent miscarriage care** |
| --- |
| “If you go down to *Hospital 7*, *Hospital 7* you walk in, it’s absolutely gorgeous. There’s a lovely reception area. They have a medical assessment room. They have a lovely outpatient reception area. And you walk in, and you walk down then, and you go up to the maternity ward, and it’s like Fort Knox. It’s awful. It’s absolutely awful. The structure is old. The walls, paint chipping off the walls. It is appalling. Absolutely appalling. And that’s where there was no director of midwifery. And they’re still fighting, but the money has all gone into other areas. And they’ve a strong voice there from the clinical director of medicine who has got all this other capital funding for what is required. So that’s a very good example of not having the right people at the executive table to advocate for the funding required.” (AGM2) |
| “If the topic is political, you will get the attention. And, unfortunately, sometimes the only way to get the attention is actually the media. And that is the strongest voice that we actually have. Women have used it, and have used it very successfully. Unfortunately, it shouldn’t be that way, but it has turned things for women… So our voices are being heard out there, and this is the result of the *Case 3* and the *Case 4,* and all those people. They have put women’s health out in front, and that’s good…… You have to. It does work. It’s an awful thing to say, but it does work. And that’s the way the system responds. That’s the way the HSE [Health Service Executive] responds. It’s a response to the next political tornado that’s coming down and that’s the reality you know… They throw money to try and cover it up, and they don’t want to hear about it, and they try to cover it. And basically that’s what happens.” (AGM2) |
| “I suppose in fairness to kind of senior management in hospitals, they’re usually put to the pin on their collar to maintain existent services. So if you’re going to start a new clinic, you have to put a midwife into it. You have to allocate a doctor. So they don’t see it as a priority. And again, one of the problems in medicine, and in our specialty, is the physical always gets prioritised over the psychological… So I suppose maybe opposition might be too strong a word, but there’s a lack of enthusiasm, and a lack of understanding about what for the women themselves is a very important support.” (AGM5) |
| “We’ve a problem in this country I think when we have guidelines. You’ll have people enthusiastic about guidelines. You then will have, you’ll have a national guideline in some areas, but if people don’t necessarily - if they weren’t part of it, or they’re not aware of it, they’ll kind of decide, you know, it’s not binding, you know, and it’s only a guideline. You then will have different hospitals decide they’ll have local guidelines. And I think that’s really poor because I think standards overall like, as much as possible, you should, if you walk in to whether its *Hospital 4* or *Hospital 1,* have a similar standard. It may not be a perfect standard in Europe, but you should have a similar standard in Ireland and that should be, and I don’t think that exists now.” (AGM6) |
| “… I’d like to see the [recurrent miscarriage] service being provided here to be truthful because I think it’s difficult having to try and have different facilities. I mean it’s clear that not every woman has that access to transport or to finance. And then of course there is a waiting time. There’s anxiety around it; if we had the services here obviously it would be a better experience for women. But again, we have to look at the cost implications and what the degree of demand is, and I suppose I’m not sure that we have that data to actually say, you know, we have quite a significant portion here, maybe we should be looking at this as a real service development for women here in the region.” (DOM2) |
| “Because I don’t think there’s anyone leading out on it. You know, I think it’s like everything. If somebody has a particular interest in an area, you’re going to see it develop and thrive. And, you know, I think that’s been absent here in the true sense. It’s not that there isn’t an interest but as in I’m going to run with this and I’m going to get… You know, I think that level of enthusiasm hasn’t been here in this specific area. So I think there is probably a movement on that, and I think that would be great to see that starting to take shape.” (DOM2) |
| “I don’t see any objection from the other consultants in this service [to the recurrent miscarriage clinic]. Women kind of put it out there, but we’re more quietly doing this and there hasn’t been any objection or anything like that. I suppose once we get numbers up a little bit better, a little bit more, that’s when I might put it out there, as in, we are going to do this.” (OBGYN1) |
| “…I would love to be more involved in a specific pregnancy loss clinic… Now I have discussed it, because I gave a presentation online of governance on the bereavement side and I did kind of plan for *Hospital 5,* and I did bring up about a pregnancy loss clinic, but it wasn’t taken kind of on. But you know that’s probably because I presented it.” (BM1) |
| “So even if you looked at the consultants within just our unit only I guarantee you if you ask some of them about how they’d manage things they’ll be different straight away you know. They aren’t all the same guidelines or criteria.” (BM2) |
| “But because there isn’t a named recurrent miscarriage management system named. It’s not overt. It’s there. It exists. They will be managed. But there is an element of hopefully at the end of that. Because it’s very easy. I mean even what I have said it is very easy. Can you not see how huge volumes of people who don’t connect can so easily fall through. And while prescriptive is not a good fit for everybody I think that when you have people… Our staff rotate through different areas so we don’t necessarily have antenatal gynae staff. We do have a more permanent and more fixed early pregnancy unit staff but they also rotate out. So if every woman who has one miscarriage this happens, two miscarriages. And these women then can opt out of that rather than having to opt in. I feel that it is easier to opt out, to choose not to go down this path, to choose not to have all this investigation if you can make that informed decision, if you know what the options are rather than having to fight to get your results, having to find the person who can… And absolutely. It does happen. It does happen very smoothly, but I think with huge holes in it.” (BM4) |
| “For a process that is consultant-endorsed or consultant-advocated there is huge buy-in…… the encouragement that they would get would be huge within something that is advocated for. It’s not so much that there is active resistance [to efforts from others, including nurses and midwives]. It’s more a passive...” (BM4) |
| So yes, getting the buy-in from the consultant like *Consultant* who is very enthusiastic is good, but what we need is from higher up funding recognition for this cohort of women I think, and get that built in.” (BM7) |
| “My line manager of course and having management being supportive is actually really, really, really important. I can’t stress it. It’s very important to have management buy-in for bereavement care in all maternity hospitals because people cannot know what we do and sometimes I feel that, and I feel that people don’t realise all that is involved. It’s very hard to quantify that support you give to somebody, a family, a couple and all the little splinters that come off that session could be a little pathway there and that then could be done. And all the multidisciplinary communication that may be involved for these women and the couples, you know. So management buy-in, and management awareness of what we do, for us here has been very important…… Because a lot of it is support consultations, stuff that’s done in the background, coordinating. It’s difficult to capture that activity you know.” (BM8) |
| “Now the consultant we have, our lead consultant is very good, and he has been saying we should be getting rid of some things in the hospital, and we should be priority. Managers are not listening, you know, and now, they need to start listening. Like they need to… really pull together and just get on with it …... We have meetings every three months, and we try and push things and... *Laughs* It’s forwards and backwards. You get excited one day, and the next thing you’re told ‘no’, you know. But anyway, you get used to it.” (M1) |
| “I think if there was a national standard. I know I’ve had consultants turn around and say to me that’s only a guideline and, yes, I see the guideline, but this is what I’m going doing here. And you’re kind of going okay, grand.” (S2) |
| **Sub-theme 3.2 \| Evidence-based service change/delivery** |
| “I suppose a very defined pathway for parents who are experiencing recurrent miscarriage where it’s very clear what they can expect and what we need to give them and this would be evidence based of course taking into account the clinical needs and whatever.” (AGM1) |
| “I think everything with medicine, obviously there’s a balance. But we do see some people then from fertility clinics been given things like intralipids, immunoglobulins, and there are definite risks with those. And you think… And they’re being charged quite a bit, and it’s a scam as far as I can see. I really think… it’s not right. And in some ways maybe we’re complicit, because we don’t necessarily say… make a big enough fuss, just, you know, role our eyes.” (AGM6) |
| “So, of the 30% of women who would be basically in the unexplained kind of criteria, I do talk to them about empiric treatment with aspirin and progesterone. And I explain to them about the lack of evidence really in terms of progesterone, and somewhat evidence I suppose of benefit of aspirin, but also the kind of safety data behind it as well. And we go back then in terms of the pattern of how their miscarriages happened to sort of have a feel on whether, what is maybe likely to help and what is less likely to help. And I also kind of emphasise as well at that point that the majority of miscarriages happen because of chromosomal abnormalities, in fact the aspirin or progesterone is not really going to help from that point of view... but there are other aspects. A lot of the times I find that women with unexplained miscarriages do tend to opt for both empiric treatment, but realising that it may or may not make a difference for their pregnancy.” (OBGYN1) |
| “Like everything, just explaining why it’s not necessarily in their interest. And then they’re in an environment where if you Google the whole thing, you will be, before you know it, on route to a private clinic who will investigate you with a lot of non-evidence-based tests. And of course people, they love tests, you know. People like investigations. So we have that confounding factor in the background where private clinics will do it, but the public service won’t you know, so they see this dilemma. But if you actually explain to them the rationale, you know, that we stick with the evidence and first do no harm, then I think people appreciate it. So yeah. And sometimes you can sound like a broken record, you know, when you’ve had a few queries about people, and oh would you not see her. Then it can be a bit tough. But you just have to just I think, keep reviewing the evidence, you know.” (OBGYN2) |
| “I think definitely having strict criteria [for a recurrent miscarriage clinic], and having leadership of the clinic from someone who is you know strict but approachable and can... Even if it was maybe a midwife in charge of it who you know can relate to. And I suppose a streamlined service, the good thing now with the electronic chart is that you can do referrals on that. So rather than a piece of paper that could potentially get lost in admin it’s a record. You can see it’s been ordered. So a referral pathway like that. I suppose not having a long wait, so having a clinic you know resourced enough that it can be held frequently and see appropriate numbers of patients.” (OBGYN4) |
| “I think really the most important thing is having a structure and a pathway for them. You know that this is the care and that its standardised and that there’s senior input and that they’re investigated you know in a standardised way that everybody has the same basic investigations done, that the investigations are followed up and that the results are interpreted by somebody who actually knows what they’re talking about… And it’s the supportive care.” (SPR2) |
| “To me the process is hugely frustrating because there is a phenomenal "it depends". I’ll chase the results. I will contact. It is not… You know I can’t say after four weeks your histology report will be back. But we won’t see you unless there’s something significant within that that we can treat and sort out. We will consolidate all your results which will be three to four weeks after the thrombophilia screen. There’s still even at that stage and "it depends" part to that because it depends on the consultant input, it depends. Because there isn’t a clear idiot proof definition of who should be seen when and where with what results. So I may be seen after my histology results are back because my consultant feels you know or because I’m gobby and keep ringing the secretary and eventually… But somebody else may not be seen until eventually after all of these things are put together you know.” (BM4) |
| “Well I suppose more universal kind of, more universal guidelines in that area. You know, maybe more publicly accessible, or, within our units, so we’re kind, all doing, universal kind of care, so we’re all doing the same thing, rather than, you know, kind of, we do this, somewhere else does something else, you know. It’s to make sure that there’s more uniform kind of guidance around it, I think. And maybe that’s something that we could tighten up like. So, you know, our pregnancy loss clinic should be the same as, say the one in *Hospital 2*, you know. You know, that we’re doing the same things, that we’re doing, you know. And that we’re updating it according to best practice, evidence-based practice. You know, that we’re all working together, for the same thing.” (BM5) |
| “Sometimes some of the consultants here, you see again it’s not always consistent, some consultants will give aspirin or progesterone. And that varies. And then you’ll have one of the other consultants who’ll actually say there’s no documented evidence to support adding in progesterone, you know, we’re not doing it. Grand, fine. You know so that’s hard for patients as well.” (S2) |
| “But as much as they could do we were still waiting on stuff and of course I got pregnant again. Now I had been prescribed Aspirin and Clonfolic and really they didn’t feel there was anything else. I’m healthy. There was no reason… They had given me that [folic acid prescription]. Once I had gone into the clinic very fast. The high Clonfolic. There is no harm in going on these, but I don’t want to prescribe you anything different you know because of your history. You know she said we do feel its just bad luck. And everything has come up like that that its bad luck. I suppose if they had said to me oh it’s because you need to take this or you need to take that or if it was a definite answer. I suppose I kept asking for that and it wasn’t there.” (PW2) |
| “Where like in one way it’s good they don’t throw all the medication at you and go, ‘look, here’s everything’. And that’s what Fertility Clinic 1… Now that I’ve gone to Fertility Clinic 1 again, they wouldn’t give me steroids this time round because when they had done the tests, they were saying my autoimmune came back, that I didn’t need it compared to the first time. So they were saying we’re not gonna recommend steroids for you and I don’t know, I got something else. I don’t know what it was. But so, in the hospital, they were just trying. And it might be just one thing in the missing link.” (PW7) |
| “I kind of got to a stage up there [in the private fertility clinic] where I felt like I was a little bit of a guineapig you know. The amount of investigations, and they were getting a little bit more and more I suppose out there. Like there was one consideration that he [consultant] wanted me to trial ADHD medication because of the inflammatory factors within my body maybe that might be reacting and it’s not known and maybe this medication will calm all that down. But again, he had done a test on that and I didn’t meet the criteria, but he said he’d try it anyway. And then he was talking about sending me out somewhere in eastern Europe to get some specific blood test done and I just kind of said no. That’s it now. I’m not coming back here...” (PW12) |
| “Basically we were given a prescription as well for subsequent pregnancies for progesterone folic acid and aspirin. And *Consultant* was clear on that call. She said look there’s not great evidence for these making a difference but there’s no harm. And it’s almost like I think her words were something along the lines of kind of it would be silly to do nothing, we might as well, you know… To be honest that wasn’t great for *Wife.* And just mentally psychologically I think there might be a bit of a placebo affect if anything. It just feels good for me and *Wife* now that we have that, okay you’re doing something more.” (PM4) |
| **Sub-theme 3.3 \| Funding and resource constraints** |
| “… having a good recurrent miscarriage service means that you have access to geneticists and genetic testing and as I say that’s not always easily done in Ireland.” (AGM4) |
| “I just think that they should invest in women. Our government should invest in women full stop and give us the care we need. And I think recurrent miscarriage should be given at least the same benefits as abortion in the country. And I do think that more testing should be done, and more treatments should be offered in the public health care system. And I don’t think you should be transferred from a public health system to IVF for anything, because I think that should be covered within our health system…… Yeah. It shouldn’t be done. Yeah. And I think the drugs should be available on the drug payment scheme.” (PW11) |
| “I absolutely wished that they were [further options] available in the hospital. Like I think… anything like this that means you don’t have to go somewhere else. Like for me it was coming from like *County 3* back to *County 1* I was always much happier and felt safer in *County 1* and better received. And I think with the fertility side of it, it is definitely something that I wish the hospitals could develop more of an area of expertise in. You know, I suppose *Bereavement Midwife*’s support was probably the main reason that I would have stuck with *Fertility Clinic 1*. Because I had the pregnancy, then I had the loss and then it took me nearly twelve months to go back to them again you know …… *Bereavement Midwife* would have definitely said to me at one point you know there isn’t really much more that we can do in terms of testing. She said I can get you another appointment with *Consultant* if you feel like that would be helpful but she said, ‘honest’ she said, ‘I can talk to her and I can find out what would she think’, and she did that. But you know had there been more access to further options in the hospital I absolutely would have stayed in the hospital.” (PW12) |
| “Like infrastructurally we’re in a really old building. It’s really terrible. And where we used to see people, it just was awful, very grim. So they [bereavement midwives] have, you know, little by little, gotten a bunch of money off you know different pots, and they have redecorated the counselling room now, you know, so it’s beautiful. It’s small but it’s beautifully painted and there’s lovely flowers painted on to the wall, and there is dim lighting, and there is, you know, it’s just those sort of touches.” (OBGYN2) |
| “I think, you know, an investment in pathology, pathology labs, so that they have some counter-top cytogenetic evaluators, they can give a fair answer to miscarriage number one, not to mention number two or number three. I think every pregnancy loss should, it would be lovely to have the option of investigating every pregnancy loss, from a chromosomal cytogenetic, from a fetal aneuploidy point of view.” (OBGYN2) |
| “200 per person [for parental karyotype testing, paid by couple]. So I send it off to *Lab 2* in *City 1,* and that’s what they charge. So there is no, I suppose profit that goes to *Hospital 3*. It’s purely going to the transfer and the lab over in *Country 2*. Because, like a number of years ago, I used to send the samples to *Hospital K,* but you could be waiting up to a year to get a result back… It’s too long for patients. Now there are some patients who would come from around the country. Like down the country some other, for some reason, *Hospital 7* and *Hospital 16* seem to be getting the quicker results from *Hospital K,* so I’d say to them go back to that hospital and do it there. But that seems to have all stopped now as well, so I pretty much send them all now to *City 1*. [I: Okay. And what’s the waiting time like on tests there then?] Only five weeks.” (OBGYN3) |
| “I suppose patients tend to pin a lot of their hopes on this [karyotype testing], that its gonna give them answers. But chances are it’s not really going to be that informative, and often can open up a whole other can of worms in relation to, you know, if there is a genetic abnormality, then do you go on and test the parents and, you know, what else are you going to find that probably has no implications in their reproductive futures. And may involve referring to a geneticist which is a whole other problem in Ireland with the long waiting lists for that.” (OBGYN4) |
| “…the two families that were involved, did their fundraising and they got local painters/decorators. They furnished the room and everything like that. They got a lovely painting of a dragonfly off a local artist and we got that put up. And, it’s just, it’s a lovely room now…. You know, it’s something that we’re very proud of to have now, you know. It’s just great to have it. Nobody wants to be in it obviously, but it is something that’s you know a huge resource for us at the moment.” (BM5) |
| **Sub-theme 3.4 \| Societal silence around miscarriage impacts on how it is perceived, prioritised (or not) and experienced** |
| “But I think as well, a lot of that is to do with it’s a societal thing… You know everybody tells you oh miscarriage is so common, and you’re almost expected to just get up and get on with it.” (PW1) |
| “It annoys me to be honest because they’re only ever on the radio after they’ve had their child, you know. I’ve a child as well so I’m not the same as somebody who hasn’t had a child. you know. Like there is a difference. I’m sure maybe I’m as upset as they would be, but I just don’t think it’s the same. And I think people on the radio are always on the radio when they have children already beforehand, or they have a load of children afterwards, and it’s all fabulous. And I don’t like listening to it. I want to hear somebody on the radio who had five miscarriages and they’re 39. That’s what I’d like to hear, you know. And I think that’s horrible and it’s probably human nature that you get comfort if somebody is worse than you, but it’s just the way it is like. Honest to God that is the way it is, you know. But I think it is good that it is on the radio. I think it is overall its good. I wouldn’t want it not to be on the radio. I think it’s good especially for men, because we know what men are like.” (PW4) |
| “I think people don’t understand you see… I think lots of people just go, ‘oh well at least you’ve one child and you’re fine, and at least you can get pregnant’. And it’s kind of dismissive. I don’t really talk about it really to be honest because, you know, I think people just feel they have to give a positive remark back, and then that’s it. People don’t really want to engage with it. It’s not really a topic that people want to talk about.” (PW5) |
| “I suppose I was cautious, because being older I guess, when I was trying for a child, you know, being conscious of my age. Most people around me, my own age, friends had fertility issues and had miscarriages. So then I never thought it would be complete plane sailing, but I didn’t expect, you know, the number of losses. Yeah, so I suppose it wasn’t like a complete surprise. Well it was I suppose. I had my first loss at twelve weeks, not feeling the heartbeat at eight and a half weeks. Most people I know who had losses had them maybe at five or six weeks or whatever. So I suppose I knew that things could go wrong, but I suppose I didn’t necessarily expect it to go wrong for myself.” (PW5) |
| “I have found the most support has been someone who has gone through it and understands. Even telling people when you’re pregnant, someone who’s had a miscarriage knows not to jump ahead too much. She has just been really good. And yeah, there’s another person that I was walking with and like I knew she had a second trimester loss, and I didn’t realise after that that she had a few miscarriages after that. But again, it was through talking. But mostly I feel people don’t talk about their miscarriages until they have had their kids and are done with their kids.” (PW7) |
| “I haven’t told even my family, or my sisters, or my mother. You know I’m the youngest of four girls, and neither my mam, nor any of my sisters, had a miscarriage. And they don’t understand it. My mam has told me before, you know, and she’d be very religious, and, you know, she was like, ‘oh well, maybe it’s just God’s way’, and ‘would you not just stop’. You know, I know she’s trying to support me and protect me, so I haven’t told them an awful lot.” (PW11) |
| “… people talk about, you know, the taboo like, you know, related to miscarriages, and how lonely it is. Like you actually couldn’t… I don’t think that captures it enough like. I think it’s probably one of the hardest things you’ll ever go through.” (PW12) |
| “I felt like all of a sudden no-one ever spoke about miscarriages ever, and now all of a sudden they’re so normal. Oh yeah, like 25%, one in five or one in four. Like oh but it’s so common, sure it’s very normal, it’s very normal, it’s very normal. Well, you know, it doesn’t feel normal. You never kind of gave us a heads up at any stage.” (PW13) |
| “Because of the previous one [miscarriage], I knew before I even went to the hospital that it was gonna happen really. So it wasn’t as, not upsetting, it wasn’t as much of a surprise.” (PW14) |
| “I think that’s a big change too, even for our first miscarriage, when my brother was a bit old-fashioned, you know, you know, or when I told my mum, you know, who is very old-fashioned. Well she actually died this year. But she wouldn’t say it, she’d say ‘oh, don’t tell anyone that’, you know, ‘that’s between you and no-one, you and God’ like. But yeah, it’s just different. It’s changing I suppose. But I think it’s changing because of the likes of me, you know, who are talking about it, and just being a bit more open, and kind of breaking the taboo of it.” (PM1) |
| “I suppose it’s not something that we think about until you actually have to go through it. Yeah, it’s not right. They should be telling. They should be teaching this in school like with sex ed and everything.” (PM2) |
| “And the other thing that I always feel bad about is sometimes they’re not aware or not prepared for the fact that even if they go down the road of fertility treatment that you can still miscarry. It’s very cruel. And a lot of this is lack of information you know. Because they think well if they’ve had miscarriages… A conversation I’ve had with many women having had two or three miscarriages and they’d say so will I go to the fertility services. And I’m like but you’re not having the problem conceiving, the problem is staying pregnant. And for whatever reason that’s happening. You know there is a difference in how it is described. And they’re not prepared for that. So there is a lot that women don’t know.” (BM2) |
| “Nobody expects to have miscarriages. Nobody expects to have multiple miscarriages. So like I do find that some couples need a lot of support, because it takes over their lives.” (OBGYN3) |
| “I suppose it’s an expectation that if you get pregnant, you’ll have a baby. And I’d like there just to be a little bit more awareness of not every pregnancy is going to end up with a baby... Women themselves find it very hard to talk about it. It’s always, always the elephant in the room, you know. Yeah. So I suppose in an ideal world a little bit more input on you know women’s health in general in secondary schools, but focusing on pregnancy.” (PMH1) |
| “I think that women in particular can be very marginalised, and often it’s their first experience of health care. And, you know, physically they’re fine, but it can be very demoralising in terms of your person, and you know what you maybe saw for yourself, and what you thought would happen in your life. You know, a lot of people kind of put a lot of, you know, they have a lot of beliefs about, we’ll be married at 25, we will have three children, they would all pass their leaving cert, they will all go to the same university as me, and my husband will be lovely or whatever. You know what I mean. And this is the first. Maybe this is the first time that your life plan is derailed.” (GDOM1) |
| “I think it’s always surprising how shocked people are that they would have a miscarriage at all. You know, so I always tell people when they come for their first antenatal visit, you know, know that it is very common so really until you reach twelve weeks you can’t relax that much, you know, and don’t be telling everybody and, you know, letting work know or anything like that because you know one in five women will have a miscarriage in the first twelve weeks, you know.” (GP2) |
| “It’s how do we do it [educate young people in school], without the fear element?” (PMH1) |
| “But I sometimes wonder do people forget about recurrent miscarriage? I don’t know, is it something to do with first trimester loss as well. Again, this is only anecdotal now in my own experience talking to people. But sometimes people, they don’t value, that’s the wrong word now, but they don’t have the same credence to first trimester loss as they might do to a second trimester loss, or a stillbirth and neonatal death…… there aren’t very many support groups that I can see specifically for recurrent miscarriage which are a very particular group of people, you know.” (AGM1) |

Note: AGM: National Administration, Governance & Management, BM: Bereavement Midwife [Clinical Midwife/Nurse Specialist in Bereavement and Loss], (G)DOM: (Group) Director of Midwifery, GP: General Practitioner, M: Midwife, OBGYN: Consultant Obstetrician/Gynaecologist (Hospital-based), PM: Man who has experienced RM, PMH: Perinatal Mental Health, PW: Woman who has experienced RM, S: Sonographer, SPR: Specialist Registrar.
