# Supplementary material for: Views of knowledge users on recurrent miscarriage services and supports in the Republic of Ireland: a qualitative interview study
Source: BMJ Open. 2025 Apr 10;15(4):e094753. doi: 10.1136/bmjopen-2024-094753 (PMC11987160; doi:10.1136/bmjopen-2024-094753)
Supplement: Supplementary file 8 [file bmjopen-2024-094753.reviewer_comments.pdf]

PEER REVIEW HISTORY

BMJ Open publishes all reviews undertaken for accepted manuscripts. Reviewers are asked to complete a checklist review form and are provided with free text boxes to elaborate on their assessment. These free text comments are reproduced below.

ARTICLE DETAILS

Title (Provisional)

Views of knowledge users on recurrent miscarriage services and supports in the Republic of Ireland: A qualitative interview study

Authors

Marita, Hennessy; Dennehy, Rebecca; Matvienko-Sikar, Karen; O'Sullivan-Lago, Ria; Ui Dhubhgain, Jennifer; Lucey, Con; O'Donoghue, Keelin

VERSION 1 - REVIEW

**Reviewer**	**1**
**Name**	**Bailey, Sarah**
**Affiliation**	**University of Southampton, Obstetrics and Gynaecology**
**Date**	**12-Nov-2024**
**COI**	**None**

BMJ open review

Thank you for giving me the opportunity to review this comprehensive paper which aims to explore the practices and experiences of those with recurrent pregnancy loss and those involved in the delivery and management of care for this patient group. This is an important area of research and one that I am particularly interested in, and I agree it provides timely and important information.

General comments

I have enjoyed reading this paper, however, my overall comment is that it is extensive, and I would suggest, needs to be more concise – the paper is long and I feel that it loses its impact in places because of this. The challenges for this paper are to present findings and to a lesser degree, the discussion in a more succinct manner, this would provide greater clarity of your work.

My main comment is that the number of interview quotations used is vast and whilst I understand that their purpose is to illustrate, support and strengthen the interpretation and findings of your study, for me, there are too many and again their impact is lost in their large number. Furthermore, the quotes aren't contextualised, it is left to the reader to work out whether they are made by a service user (woman? partner? Number of miscarriages? Or service provider?) and this makes it more challenging to understand their applicability. Unless I have missed, it is not clear what the abbreviations mean after the quotes e.g. PW2??? – please make clear what these abbreviations stand for. I would suggest the use of significantly less, contextualised, quotations. As a qualitative researcher, I completely understand the wish to share all the wonderful quotations that your participants have so generously shared but

consider using the ones that really bring the content of your paper ‘to life’ – perhaps consider adding the rest as a supplementary file.

Introduction

Generally, well written, providing background to the study and identifying main aims / objectives. I was particularly interested to read that you are exploring health professionals’ experiences of caring for couples with recurrent miscarriage.

Methods

Line 110 – 112 – please clarify what you mean by power operates at multiple levels and how this impacts on your study.

Setting and participants

Purposeful sample needs more clarification. On what characteristics and why were participants purposively selected? Or is this a convenience sample?

Where and how did you recruit the women and partners from? I read that they were recruited through professional, collegial and support networks, but is there any more detail on this? I am partly asking this as it was over two years since some had experienced their most recent loss and so how did you access these service users? Were they patients?

Where and how did you recruit health professionals? How many institutions were they from?

How did they consent to participating in the study and were they given time to consider their participation?

Data generation

Is it possible to give an example of some of the questions in the topic guides (maybe add a table or a supplementary table). What were these questions based on?

Data analysis

You say you used reflexive thematic analysis - this section gives a limited description of the qualitive data analysis employed, but I can see you have added this information into supplementary file 2 – please direct the reader to this. This information is important to aid reproducibility.

Results

As mentioned previously, overall, this is a long section, is it possible to be more concise throughout?

Can you clarify the over arching theme – is this the over arching theme of what study participants wanted?

Lines 252 – 255 – v interesting that some health professionals voiced concerns about how specialisation of RM care can limit availability of trained staff – do you discuss this finding in discussion section?

Theme 1 (lines 224 – 303) well written and interesting concise findings.

Theme 2 (lines 305 – 430) this is quite a long section and might benefit from re-working so more succinct.

I wasn't sure how sub theme 2.4 (Recognising and meeting needs of men/partners) fitted with in the theme of 'dedicated space and time'.

Theme 3 (lines 438 – 546)

Some repetition in starting sentences e.g many health professionals (line 440, 447, 458)

Sub theme 3.2 (lines 477 – 502) This was a very interesting section and raised the question about non evidence-based investigations and treatment often available in the private sector, similarly the use of 'empiric' treatments. I'm hoping this will be re visited in discussion section as this is a real issue / dilemma for health professionals. How does this sit with the 'graded approach' as advocated in your paper and the Lancet series?

Sub theme 3.4 – (societal silence around miscarriage) this too is a relevant and important finding, but please clarify how this fits in the main theme of 'Dedicated funding and support'

Discussion

Overall, I thought this section was well written, but could have been more concise. A few comments as per below:

(Line 554) – why does your analysis support the model of care proposed in Lancet Series? Please clarify in the discussion.

I'm unable to see that a few of the really interesting findings were discussed, in particular about how we manage expectations of patients/service users as in my clinical experience this is a major consideration. Should we offer empirical care and treatment or stick to the guidelines? What about the contradiction and differences between private care and nhs or general care as offered in Republic of Ireland?

I hope the above comments are helpful.

**Reviewer**	**2**
**Name**	**Zeghiche, Sabrina**
**Affiliation**	**Université du Québec en Outaouais**
**Date**	**15-Feb-2025**
**COI**	**None**

This is a very well written, very well structured and very clear manuscript. it was a pleasure to read. It is exhaustive and very informative. The topic of RM is indeed understudied and this manuscript adds valuable information to this topic.

I have very minor comments :

- Method section : it would be useful to know exactly what qualitative research design/approach was used. As Qualitative research implies necessarily a constructivist paradigm, it is not sufficient to know exactly what appraoch was used to guide this study.

- Analysis : The data from the women, their partners, or the different health professionals were analysed together (in an indiscriminate and indifferentiated manner). While this may have been justified, it is not addressed in the manuscript and (in my opinion) can entail some limitations. Conflating all the point of views can remove the nuance and complexity of the data material as well as the contradictions and conflicting points of views.

- Background and analysis : this manuscript aims to shed light on the specific experience of RM but I think it would be useful to highlight what makes this experience unique, compared to a unique instance of miscarriage. This should be reflected both in the contextualisation and in the analysis sections.

- Some precisions to be included: (see attached document)

**** **The reviewer provided a marked copy with additional comments. Please contact the publisher for full details.******

VERSION 1 - AUTHOR RESPONSE

REVIEWER #1: Dr Sarah Bailey, University of Southampton

Comment	Author response
Please see the attached file BMJ open review.pdf.<br><br>Thank you for giving me the opportunity to review this comprehensive paper which aims to explore the practices and experiences of those with recurrent pregnancy loss and those involved in the delivery and management of care for this patient group. This is an important area of research and one that I am particularly interested in, and I agree it provides timely and important information.	Thank you for your time and valuable perspectives.
General comments	
I have enjoyed reading this paper, however, my overall comment is that it is extensive, and I would suggest, needs to be more concise – the paper is long and I feel that it loses its impact in places because of this. The challenges for this paper are to present findings and to a lesser degree, the discussion in a more succinct manner, this would provide greater clarity of your work.	Thank you for this feedback. We have edited the results and discussion accordingly. All edits appear as tracked changes.

My main comment is that the number of interview quotations used is vast and whilst I understand that their purpose is to illustrate, support and strengthen the interpretation and findings of your study, for me, there are too many and again their impact is lost in their large number.	Thank you for this feedback.<br><br>We noted in our cover letter that we were conscious that the manuscript is somewhat lengthy given the volume of data generated and to do justice to the analysis, and that we are open to making any necessary revisions, including moving the tables containing quotes to supplementary files.

Furthermore, the quotes aren't contextualised, it is left to the reader to work out whether they are made by a service user (woman? partner? Number of miscarriages?<br>Or service provider?) and this makes it more challenging to understand their applicability. Unless I have missed, it is not clear what the abbreviations mean after the<br>quotes e.g. PW2??? – please make clear what these abbreviations stand for.<br><br>I would suggest the use of significantly less, contextualised, quotations. As a qualitative researcher, I completely understand the wish to share all the wonderful quotations that your participants have so generously shared but consider using the ones that really bring the content of your paper 'to life' – perhaps consider adding the rest as a supplementary file.	Apologies, for clarity, we have added the following sentence to the introductory section of the results (pages 10-11):<br><br>"Illustrative quotes are presented under each theme, with identifiers indicating the knowledge user group (see Table 1) and participant number; for example, PW1 signifies a woman with lived experience of RM."<br><br>We have selected some quotes to include within the main body of the manuscript and have moved others to supplementary files. This has reduced the length of the manuscript.
Introduction	
Generally, well written, providing background to the study and identifying main aims / objectives. I was particularly interested to read that you are exploring health professionals' experiences of caring for couples with recurrent miscarriage.	Thank you for this positive feedback.
Methods	
Line 110 – 112 – please clarify what you mean by power operates at multiple levels and how this impacts on your study.	We have clarified what we mean as follows:<br><br>"Throughout the study we attended to wider organisational contexts and systems during data generation and analysis, given that power operates at multiple levels to influence action around improving healthcare experiences, including power held (or indeed withheld) within and across service users, staff, teams and broader hospital and healthcare systems and structures.[39]"
Setting and participants	

Purposeful sample needs more clarification. On what characteristics and why were participants purposively selected? Or is this a convenience sample?	We note this as follows:<br><br>“A purposive sampling strategy, supplemented with snowballing techniques, was used to recruit a variety of people with knowledge and experience of delivering and/or using RM services and supports in the Republic of Ireland where–at the time of the research–RM was typically defined as three or more consecutive miscarriages.[29] Women and men/partners aged over 18 years and who had two or more
	consecutive first-trimester miscarriages (to ensure that we gained insights into the experiences of those who met the referral criteria for RM services within the Republic of Ireland and those who did not, also reflecting the varying definitions of RM internationally[42]), with the most recent loss occurring 6-24 months prior to study participation were eligible to participate. We also aimed to ensure that perspectives from various hospital groups (n=6), and tertiary, regional, and peripheral maternity units (n=19) across the Republic of Ireland were included.”<br><br>We purposively selected participants: women and men, with two and three or more consecutive first trimester miscarriages – and also people involved in delivering services – ensuring we had participants from different hospitals groups and types of maternity units.

Where and how did you recruit the women and partners from? I read that they were recruited through professional, collegial and support networks, but is there any more detail on this? I am partly asking this as it was over two years since some had experienced their most recent loss and so how did you access these service users? Were they patients?	We have added the following clarification to the text:<br><br>"Participants were identified and recruited through the professional, collegial, and support networks of the research team and RE:CURRENT Research Advisory Group; this included recruitment of people known to the research team or Advisory Group members in personal or professional contexts including hospitals, support organisations and professional bodies. Social media was used to enhance the recruitment of women and men/partners with RM in later phases of the study. We ceased data collection when there was sufficient depth and variation in the data.[43]"<br><br>Women and partners were recruited via the research team and Advisory Group's networks (e.g. current or former patients, or via support groups) as well as via social media.
Where and how did you recruit health professionals? How many institutions were they from?	Health professionals were similarly recruited via through professional, collegial and support networks, and from across the country.<br><br>We have added the following as a footnote to Table 1:<br><br>"The 42 people involved in the management/delivery of services and supports were based at 29 different institutions or organisations at the time of interview. This included 14 of the 19 maternity hospitals/units in Ireland. It should be noted that some had experience across a range of maternity hospitals/units, including as part of their training."
Data generation	
Is it possible to give an example of some of the questions in the topic guides (maybe add a table or a supplementary table). What were these questions based on?	We have added the topic guides to the supplementary files, and updated the text accordingly:<br><br>"Two topic guides were used to structure discussions, one specific to people with lived experience, and one for those involved in RM service and/or support provision (see Supplementary Files 2 and 3, respectively). These were informed by the extant literature and discussions with the RE:CURRENT Research Advisory Group.
Data analysis	

You say you used reflexive thematic analysis - this section gives a limited description of the qualitive data analysis employed, but I can see you have added this information into supplementary file 2 – please direct the reader to this. This information is important to aid reproducibility.	We have directed readers to this file, as follows:<br><br>"Using reflexive thematic analysis, a rigorous iterative process of data familiarisation, data coding, theme development and revision, refinement and write up, was undertaken;[37] see further details in Supplementary File 4."
Results	
As mentioned previously, overall, this is a long section, is it possible to be more concise throughout?	We have read through the entire manuscript – focusing particularly on the results section – and have made edits (changes tracked) to reduce the word count and make our points more concise.
Can you clarify the over arching theme – is this the over arching theme of what study participants wanted?	We have added clarification to the text, as follows:<br><br>"We generated one overarching theme from the data, which captures what RM services and supports should encompass based on participants' accounts: "A standardised, dedicated, and adequately resourced and supported service in which women and men/partners experiencing RM are offered appropriate, individualised, timely and accessible care and support–this should begin following the first miscarriage and follow a graded approach"."
Lines 252 – 255 – v interesting that some health professionals voiced concerns about how specialisation of RM care can limit availability of trained staff – do you discuss this finding in discussion section?	Thank you for highlighting this point; we have now included it in the discussion around dedicated staff as follows:<br><br>"Though only raised by a few health professionals, the perceived negative impacts of
	specialisation in RM need to be addressed and basic training for all provided; this should include education around RM clinical guidelines."
Theme 1 (lines 224 – 303) well written and interesting concise findings.	Thank you for this feedback.
Theme 2 (lines 305 – 430) this is quite a long section and might benefit from re-working so more succinct.	Thank you; we have edited accordingly.
I wasn't sure how sub theme 2.4 (Recognising and meeting needs of men/partners) fitted with in the theme of 'dedicated space and time'.	We have added the following clarification to this sentence:<br><br>"Participants spoke about how the needs of partners were under-recognised and unmet within services, highlighting the importance of dedicated space and time to address these needs."

Theme 3 (lines 438 – 546): Some repetition in starting sentences e.g many health professionals (line 440, 447, 458)	Thank you for highlighting. We have reworded the introductory lines to these three paragraphs, as follows:<br><br>"Health professionals noted…"<br><br>"Many – particularly doctors and CMS-BL – stressed…"<br><br>"Those who championed service improvements spoke about…"
Sub theme 3.2 (lines 477 – 502) This was a very interesting section and raised the question about non evidence-based investigations and treatment often available in the private sector, similarly the use of 'empiric' treatments. I'm hoping this will be re visited in discussion section as this is a real issue / dilemma for health professionals. How does this sit with the 'graded approach' as advocated in your paper and the Lancet series?	Thank you; based on your feedback we have now included this within the discussion, as follows:<br><br>"Education on evidence-based investigations and treatments for RM is also needed, for health professionals and people with lived experience alike. The offering and use of unconventional investigations and treatments, particularly within the private sector, must be addressed. People with lived experience clearly valued explanations from health professionals, therefore providing information and support even within the uncertainty will help. Dedicating appropriate space and time within clinics – and more broadly – to communicating the unique challenges that RM presents, the limited evidence-based investigations and treatments available, and prognosis for future pregnancies may assist in managing expectations and informing decision-making. Investing in, and standardising and structuring, RM care within the health service in line with clinical guidelines and a graded model may alleviate people's perceptions that the public system could have done more for them."

Sub theme 3.4 – (societal silence around miscarriage) this too is a relevant and important finding, but please clarify how this fits in the main theme of 'Dedicated funding and support'	In the introduction to Theme 3 we note:<br><br>"The third and final theme relates to broader system issues which influence how RM, and indeed pregnancy loss more broadly, is perceived, understood, (de)prioritised and how these impact – or could be harnessed to positively impact – policy and practice."<br><br>We have edited the name of this theme, for greater clarity:<br><br>"Societal silence around miscarriage impacts on how it is perceived, prioritised (or not) and experienced"<br><br>The introductory paragraph discusses how it influences resourcing (funding) and supports:<br><br>"Many participants highlighted how the silence around miscarriage/pregnancy loss impacted on how it was perceived – by all – influencing public understanding and expectations of people with lived experience, as well as prioritisation by health service decision-makers; ultimately impacting the prioritisation, resourcing and delivery of services, and how women and men experienced pregnancy loss and subsequent care and supports."
Discussion	
Overall, I thought this section was well written, but could have been more concise. A few comments as per below:	We appreciate your feedback.
(Line 554) – why does your analysis support the model of care proposed in Lancet Series? Please clarify in the discussion.	We found that people's experiences of recurrent miscarriage were very much influenced by their experiences during their first miscarriage, hence our recommendation for a graded approach following a first miscarriage.<br><br>We have added the following clarification to the text:<br><br>"Our analysis provides support for the model of RM care proposed in the Lancet Series on Miscarriage, and the need for a graded approach following a first miscarriage.[33] In our study, people's experiences and perspectives were shaped from their first miscarriage; better meeting their needs at this point, by offering healthcare advice and support, may improve experiences and outcomes. Care can then be

	augmented following two miscarriages and three miscarriages, via nurse or midwife-led clinics and medical consultant-led clinics, respectively.[33]”
I’m unable to see that a few of the really interesting findings were discussed, in particular about how we manage expectations of patients/service users as in my clinical experience this is a major consideration. Should we offer empirical care and treatment or stick to the guidelines? What about the contradiction and differences between private care and nhs or general care as offered in Republic of Ireland?	The feedback has been useful in highlighting important discussion points as there are many insights from this work.<br><br>As noted above, we have added the following to the discussion:<br><br>“Education on evidence-based investigations and treatments for RM is also needed, for health professionals and people with lived experience alike. The offering and use of unconventional investigations and treatments, particularly within the private sector, must be addressed. People with lived experience clearly valued explanations from health professionals, therefore providing information and support even within the uncertainty will help. Dedicating appropriate space and time within clinics – and more broadly – to communicating the unique challenges that RM presents, the limited evidence-based investigations and treatments available, and prognosis for future pregnancies may assist in managing expectations and informing decision-making. Investing in, and standardising and structuring, RM care within the health service in line with clinical guidelines and a graded model may alleviate people’s perceptions that the public system could have done more for them.”
I hope the above comments are helpful.	They have been very helpful, thank you.

REVIEWER #2: Dr Sabrina Zeghiche, Université du Québec en Outaouais

Comment	Author response
This is a very well written, very well structured and very clear manuscript. it was a pleasure to read. It is exhaustive and very informative. The topic of RM is indeed understudied and this manuscript adds valuable information to this topic. I have very minor comments:	Thank you for your time; we appreciate your constructive feedback our manuscript.
Method section	
It would be useful to know exactly what qualitative research design/approach was used. As Qualitative research implies necessarily a constructivist paradigm, it is not sufficient to know exactly what appraoch was used to guide this study.	This section now reads:<br><br>"We employed a qualitative study design, underpinned by constructivism and incorporating semi-structured interviews and reflexive thematic analysis, to facilitate an in-depth exploration of the views and experiences of women and men with lived experience of RM and those involved in the provision of services and supports. Aligned with this, we acknowledge that knowledge is socially produced, and the role that researchers play in in co- creating knowledge and meaning.[37] We adopted a critical qualitative inquiry approach within our study, underpinned by social justice,[38] as we aimed to interpret people's views and experiences of RM care whilst also trying to affect change in service provision."<br><br>We hope this addresses your query.
Analysis	
The data from the women, their partners, or the different health professionals were analysed together (in an indiscriminate and indifferentiated manner). While this may have been justified, it is not addressed in the manuscript and (in my opinion) can entail some limitations. Conflating all the point of views can remove the nuance and complexity of the data material as well as the contradictions and conflicting points of views.	We have added the following justification to the data analysis section:<br><br>"During data generation and analysis we observed similarities within the data from both groupings, therefore we analysed them together to provide a multifaceted understanding of RM services and supports."<br><br>We note in the discussion that "The inclusion of a diverse range of perspectives – including people with lived and professional expertise – is a key strength of this study."<br><br>Thematic analysis involves generating common patterns or themes from the data. While the themes (and sub-themes) apply across both groupings (people with lived experience and service providers), in the discussion of these within the results section, we have noted where there are diverging views between people with lived experience and service providers, or more commonly, where one group provides additional insights into an aspect of care.

	For example, page 19:<br><br>"The need for non-judgemental, empathic communication – from the first miscarriage experienced through any subsequent pregnancies – was highlighted. This included during communications about risk factors (particularly regarding age) and advice regarding behavioural and/or other changes that participants could make to prepare for subsequent pregnancies. Health professionals often discussed this in the context of trying to 'empower' women/men, when they could often feel powerless given the nature of RM."
Background and analysis	
This manuscript aims to shed light on the specific experience of RM but I think it would be useful to highlight what makes this experience unique, compared to a unique instance of miscarriage. This should be reflected both in the contextualisation and in the analysis sections.	We have added the following to the discussion to address your initial comment:<br><br>"Negative psychological impacts, including grief, depression, anxiety and post-traumatic stress disorder, can be amplified for people with RM.[3-7]"<br><br>We have not delved further into the RM experience as this paper focuses on care experiences and opportunities to enhance services and supports, and the manuscript is already quite lengthy.<br><br>The analysis section reflects the experience of recurrent miscarriage; however, we found that this was very much influenced by people's experiences during their first miscarriage, hence our recommendation for a graded approach following a first miscarriage.
Some precisions to be included: (see attached document)	
Page 2, line 6: "RM" - Write [RM] in full (for the first occurrence)	Thank you for highlighting this; we have spelled RM out at first mention.
Page 2, line 6: "Care" - in the hospital, after discharge from hospital, short-term, long term care? Specifiy.	We have clarified as follows:<br><br>"Women and men/partners who experience miscarriage often report poor care experiences within health services around the time of miscarriage and beyond …"<br><br>We have not further elaborated as this is the abstract; we are constrained by word limits.
Page 2, line 9: "recurrent miscarriage (RM)" - See previous comment	This has been edited to RM as 'recurrent miscarriage (RM)' is previously mentioned – in response to previous comment.
Page 2, line 23: "in a graded manner" - What do you mean exactly?	By graded we mean 'graded model', where people are offered healthcare advice and support after one miscarriage, care in a nurse or midwife-led clinic after two miscarriages, and care in a medical consultant-led clinic after three miscarriages, in line with the Lancet Commission's recommendations.

	This sentence now reads:<br><br>"One in which people experiencing RM are offered appropriate, individualised, timely and accessible care and support – beginning following the first miscarriage, and following a graded model."
Page 5, line 85: "hospital environments" - Could you be a bit more specific?	We have added the following clarification:<br><br>"Recurring themes across the aforementioned literature include deficits regarding communication and information, hospital environments (e.g. shared spaces for pregnancy loss and maternity care), and supportive and follow-up care, as well as the lack of training, supports, and resources for health professionals."
Page 5, line 88: "graded model" - Could you be a bit more specific?	We have added the following detail:<br><br>"This includes offering people healthcare advice and support after one miscarriage, care in a nurse or midwife-led clinic after two miscarriages, and care in a medical consultant-led clinic after three miscarriages, in line with the Lancet Commission's recommendations.[33]"
Page 6, line 105: "qualitative study design" - There different qualitative designs. Which one exactly did you use?	As noted above, this now reads:<br><br>"We employed a qualitative study design, underpinned by constructivism and incorporating semi-structured interviews and reflexive thematic analysis, to facilitate an in-depth exploration of the views and experiences of women and men with lived experience of RM and those involved in the provision of services and supports."
Page 6, line 110: "wider organisational contexts" - Can you specify?	This section has been edited further to comments from Reviewer 1 also. It now reads:<br><br>"Throughout the study we attended to wider organisational contexts and systems during data generation and analysis, given that power operates at multiple levels to influence action around improving healthcare experiences, including power held (or indeed withheld) within and across service users, staff, teams and broader hospital and healthcare systems and structures.[39]"
Page 7, line 126: "Distress protocols" - what were those protocols	Within our protocol, if participants become upset during an interview we offer to pause and/or stop the interview. Participants are initially encouraged to contact a family member or friend for support before the end of the session. With their permission, the researcher checks-in with them within 24 hours, and provides details of support networks, and, where deemed necessary, and with their permission, they are referred to the clinical nurse/midwife bereavement specialist, or medical social worker, in the relevant maternity unit/hospital.

	Interviews were conducted by RD and MH, both experienced qualitative interviewers/researchers. Following each interview, RD contacted MH, or vice versa, and they debriefed. If distressed following an interview, they could also debrief with a senior member of the research team. If further support was required, they would be directed to counselling services.<br><br>Briefing and debriefing took place with the professional transcriptionist.<br><br>Given the need to reduce the word count of the manuscript, we have made it clearer within the text that the distress protocol is available elsewhere:<br><br>"Distress protocols were in place for participants, researchers, and the professional transcriber; these are outlined elsewhere.[41]"
Page 7, lines 131-132: "through their networks" - why limit yourself to only this channel of recruitment. Does it not run the risk of recruitment bias?	We note in the Patient and Public Involvement section that members of the RE:CURRENT Research Advisory Group recruited participants through their networks.<br><br>In the recruitment section we provide more details on recruitment, as follows:<br><br>"Participants were identified and recruited through the professional, collegial, and support networks of the research team and RE:CURRENT Research Advisory Group; this included recruitment of people known to the research team or Advisory Group members in personal or professional contexts including hospitals, support organisations and professional bodies. Social media was used to enhance the recruitment of women and men/partners with RM in later phases of the study. We ceased data collection when there was sufficient depth and variation in the data.[43]"<br><br>We used a purposive sampling strategy, supplemented with snowballing techniques, ensuring we included perspectives from various hospital groups, and tertiary, regional, and peripheral maternity units (which have different RM care structures).<br><br>Research Advisory Group members were from a range of geographical locations, organisations/services (including different hospitals) and disciplines, and were well-placed to identify potential participants (and information-rich cases) in line with our sampling framework.<br><br>We ceased data collection when there was sufficient depth and variation in the data.

	The above measures mitigated the potential for recruitment bias.
Page 7, line 142: “6-24 months” - why this time frame?	We have added the following justification:<br><br>“The latter was to allow sufficient time since the most recent miscarriage whilst capturing relatively recent experiences of services and supports.”
Page 8, line 158: “discussion” - missing period at the end of this sentence.	We have added a full stop to the end of this sentence.
Page 8, line 164: “knowledge and understanding” - was this asked also to parents? If so, can you explain what was the aim of this question?	This was asked of service providers and people with lived experience. Prompts for this topic are available in the topics guides now added as supplementary files.<br><br>We asked this question as we wanted to explore knowledge and understanding, particularly in relation to what people’s expectations of care might be and then how this compared with their experiences when they had two or more consecutive miscarriages.<br><br>This formed part of our strategy in attending to wider organisational contexts and systems to better understand how we might improve care experiences.<br><br>In practice, during interviews, people with lived experience tended to tell their story from the outset of the interview, with little input required from the interviewer. They automatically addressed areas outlined within the topic guide, with issues around knowledge and understanding and expectations around pregnancy mentioned.
Page 8, lines 166-167: “expectations of pregnancy before loss, and subsequent pregnancies” - can you explain how this relates to the objective of the study?	As above.
Page 9, line 185: “13 women” - are these 13 women all women who were pregnant and had a miscarriage and are some of them partners of women who had a miscarriage?	These were all women who were pregnant and had two or more consecutive miscarriages. We have edited the text in Table 1, as follows:<br><br>“Women who have experienced recurrent miscarriage**,** having carried the pregnancies”
Page 9, line 186: “14 of whom had living children” - Before and/or after the RM?<br><br>I see you specify this in the table below. Ignore my comment.	N/A
Page 9, line 188: “preference for a telephone interview” - Did they explain why?	They opted for a telephone interview because it was more convenient for them, e.g. because of where they were/what they had access to at the time of interview.

	This sentence now reads:<br><br>"A minority of participants expressed a preference for a telephone interview because it was more convenient for them."
Page 13, line 222: "Table 3" - I am not sure if this is a common practice in this journal but it seems a bit odd to me to have a lengthy table of quoates, instead of spreading them throughout the results section.<br><br>"(PW3)" - it is not clear what this code refers to : is a quote from a parent (woman or her partner) or from a member of staff?<br><br>"(PM1)" - same comment	Thank you for raising this. We noted in our cover letter that we were conscious that the manuscript is somewhat lengthy given the volume of data generated and to do justice to the analysis, and that we are open to making any necessary revisions, including moving the tables containing quotes to supplementary files.<br><br>We have selected some quotes to include within the main body of the manuscript and have moved others to supplementary files.<br><br>Apologies, for clarity, we have added the following sentence to the introductory section of the results (pages 10-11):<br><br>"Illustrative quotes are presented under each theme, with identifiers indicating the knowledge user group (see Table 1) and participant number; for example, PW1 signifies a woman with lived experience of RM."<br><br>We have also added a note to each of the supplementary files containing quotes (Supplementary Files 5-7) to explain abbreviations used.
Page 18, line 225: "theme" - subtheme?	Apologies, this should be 'sub-theme'; we have corrected, thank you.
Page 22, line 311: "Table 4" - same comment as for the first table of quotes	We have selected some quotes to include within the main body of the manuscript and have moved others to supplementary files.
Page 33, line 428: "acknowledge" - acknowledging?	Thank you for spotting this typo. This now reads:<br><br>"Many health professionals spoke about actively encouraging men/partners to attend appointments and the importance of acknowledging their loss and grief."
Page 34, line 436: "Table 5" - Same comment as before	We have selected some quotes to include within the main body of the manuscript and have moved others to supplementary files.
Page 49, line 621: "themselves" - missing period at the end of this sentence.	Thank you; we have added a full stop to the end of this sentence.
